# *De novo* variants in congenital diaphragmatic hernia identify *MYRF* as a new syndrome and reveal genetic overlaps with other developmental disorders

**DOI:** 10.1371/journal.pgen.1007822

**Published:** 2018-12-10

**Authors:** Hongjian Qi, Lan Yu, Xueya Zhou, Julia Wynn, Haoquan Zhao, Yicheng Guo, Na Zhu, Alexander Kitaygorodsky, Rebecca Hernan, Gudrun Aspelund, Foong-Yen Lim, Timothy Crombleholme, Robert Cusick, Kenneth Azarow, Melissa E. Danko, Dai Chung, Brad W. Warner, George B. Mychaliska, Douglas Potoka, Amy J. Wagner, Mahmoud ElFiky, Jay M. Wilson, Debbie Nickerson, Michael Bamshad, Frances A. High, Mauro Longoni, Patricia K. Donahoe, Wendy K. Chung, Yufeng Shen

**Affiliations:** 1 Department of Systems Biology, Columbia University Medical Center, New York, New York, United States of America; 2 Department of Applied Mathematics and Applied Physics, Columbia University, New York, New York, United States of America; 3 Department of Pediatrics Medical Center, Columbia University, New York, New York, United States of America; 4 Department of Biomedical Informatics, Columbia University Medical Center, New York, New York, United States of America; 5 Department of Surgery, Columbia University Medical Center, New York, New York, United States of America; 6 Cincinnati Children's Hospital, Cincinnati, Ohio, United States of America; 7 Children's Hospital & Medical Center of Omaha, University of Nebraska College of Medicine, Omaha, Nebraska, United States of America; 8 Department of Surgery, Oregon Health & Science University, Portland, Oregon, United States of America; 9 Monroe Carell Jr. Children's Hospital, Vanderbilt University Medical Center, Nashville, Tennessee, United States of America; 10 Washington University, St. Louis Children's Hospital, St. Louis, Missouri, United States of America; 11 University of Michigan, CS Mott Children's Hospital, Ann Arbor, Michigan, United States of America; 12 Children's Hospital of Pittsburgh, Pittsburgh, Pennsylvania, United States of America; 13 Medical College of Wisconsin, Milwaukee, Wisconsin, United States of America; 14 Department of Pediatric Surgery, Faculty of Medicine, Cairo University, Cairo, Egypt; 15 Department of Surgery, Boston Children’s Hospital, Boston, Massachusetts, United States of America; 16 Department of Surgery, Harvard Medical School, Boston, Massachusetts, United States of America; 17 University of Washington, Seattle, Washington, United States of America; 18 Pediatric Surgical Research Laboratories, Department of Surgery, Massachusetts General Hospital, Boston, Massachusetts, United States of America; 19 Department of Medicine, Columbia University, New York, New York, United States of America; 20 Herbert Irving Comprehensive Cancer Center, Columbia University Medical Center, New York, New York, United States of America; 21 JP Sulzberger Columbia Genome Center, Columbia University Medical Center, New York, New York, United States of America; Max Planck Institute for Molecular Genetics, GERMANY

## Abstract

Congenital diaphragmatic hernia (CDH) is a severe birth defect that is often accompanied by other congenital anomalies. Previous exome sequencing studies for CDH have supported a role of *de novo* damaging variants but did not identify any recurrently mutated genes. To investigate further the genetics of CDH, we analyzed *de novo* coding variants in 362 proband-parent trios including 271 new trios reported in this study. We identified four unrelated individuals with damaging *de novo* variants in *MYRF* (P = 5.3x10^-8^), including one likely gene-disrupting (LGD) and three deleterious missense (D-mis) variants. Eight additional individuals with *de novo* LGD or missense variants were identified from our other genetic studies or from the literature. Common phenotypes of *MYRF de novo* variant carriers include CDH, congenital heart disease and genitourinary abnormalities, suggesting that it represents a novel syndrome. *MYRF* is a membrane associated transcriptional factor highly expressed in developing diaphragm and is depleted of LGD variants in the general population. All *de novo* missense variants aggregated in two functional protein domains. Analyzing the transcriptome of patient-derived diaphragm fibroblast cells suggest that disease associated variants abolish the transcription factor activity. Furthermore, we showed that the remaining genes with damaging variants in CDH significantly overlap with genes implicated in other developmental disorders. Gene expression patterns and patient phenotypes support pleiotropic effects of damaging variants in these genes on CDH and other developmental disorders. Finally, functional enrichment analysis implicates the disruption of regulation of gene expression, kinase activities, intra-cellular signaling, and cytoskeleton organization as pathogenic mechanisms in CDH.

## Author summary

Congenital diaphragmatic hernia (CDH) is a life-threatening condition affecting about 1 every 3000 newborns. Although the role of genetics in the pathogenesis of CDH has been well established, only a handful of disease genes have been identified so far. We and other have previously shown that *de novo* variants, those carried by the cases but not inherited from parents, are enriched in sporadic CDH cases consistent with their negative effects on reproductive fitness. To further investigate the genetics of CDH, we analyzed *de novo* variants in 362 proband-father-mother trios from whole exome or genome sequencing data and identified four patients carrying damaging variants in *MYRF*, a membrane associated transcription factor that is highly expressed in developing diaphragm and heart. We then ascertained a total of 12 patients with *MYRF de novo* variants, and found they shared common phenotype characteristics including congenital abnormalities in diaphragm, heart and reproductive organs. The high rate of recurrence and similar phenotypic manifestations suggest that *de novo* variants of *MYRF* have pleiotropic effects and cause a novel syndrome. The identified new gene is reminiscent of previously identified CDH genes (e.g., *GATA4*, *GATA6*, *NR2F2*, *ZFPM*2, and *WT1*) that are also associated with other developmental disorders. Indeed, we found in our cohort more than 20 damaging *de novo* variants in genes implicated in other developmental disorders but not previously linked to CDH. The overlap was unlikely to occur by chance and can be best explained by their pleiotropic effects. We also showed that, despite the shared genetic basis with other disorders, damaging de novo variants in CDH as a whole were enriched in specific functional pathways that recapitulated our current knowledge about diaphragm development. So additional candidate genes can be prioritized based on the genetic pleiotropy and functional specificity. The findings have general implications in design and analysis in genetic studies of rare birth defects.

## Introduction

Congenital diaphragmatic hernia (CDH) is a severe developmental disorder affecting 1 in 3000 live births [1, 2]. It is characterized by defects in diaphragm that allow the abdominal viscera to move into the thoracic cavity and is associated with pulmonary hypoplasia and in some cases pulmonary hypertension. CDH can be isolated (50–60%) or associated with anomalies in other organs including the heart, brain, kidneys and genitalia [3, 4]. Despite advances in treatment, mortality rate remains high [5, 6]. A better understanding of the causative factors for CDH may inform disease prevention and treatment.

The genetic contribution to CDH has been established by familial aggregation [7], rare monogenic disorders associated with CDH in humans [8], chromosome abnormalities [9], copy number variations [10–12], and mouse models [13]. However, our understanding of the genetic basis of CDH is still rudimentary. The historically low reproductive fitness of individuals with CDH led to the hypothesis that *de novo* variants with large effect sizes may explain a fraction of CDH patients as in other developmental disorders [14, 15]. We and others have previously reported an enrichment of damaging variants in sporadic CDH patients [16, 17]. However, no recurrently mutated gene was identified in our genome wide analyses due to the limited sample size.

To continue the search for new CDH genes, we performed whole exome (WES) or whole genome sequencing (WGS) of 271 new trios. Combined with previously published WES data [16, 17], we analyzed all 362 trios. We confirmed the overall burden of damaging *de novo* variants and identified a new disease gene recurrently mutated in cases with similar syndromic features. To prioritize additional risk genes, we analyzed cross-disorder overlap and pathway enrichment. The results provide insights into the genetic architecture of CDH and suggest additional candidate genes.

## Results

### Sample characteristics

Patients were recruited from the multicenter, longitudinal DHREAMS (Diaphragmatic Hernia Research & Exploration; Advancing Molecular Science) study [11]. We excluded patients with known genetic causes from clinical karyotype or chromosome microarray or with a family history of CDH. WES was performed on 118 proband-parents trios, a subset (39) of whom were published previously [17]. WGS was performed on 192 trios including 27 without damaging variants from the previous study [17]. On average, 91% of coding regions in WES samples and 98% in WGS samples were covered by 10 or more unique reads (S1 Fig). WGS showed more uniform distribution of sequencing depth that contributes to higher power in detecting coding variants [18, 19]. For the 27 overlapping samples, 12 additional *de novo* coding variants were identified in WGS including 10 not included in the exome targets or with low depth of coverage and two that failed stringent QC filters in our previous study.

Combined with trios collected by Boston Children’s Hospital/Massachusetts General Hospital (BCH/MGH) [16], we analyzed a total 362 unique trios (S1 Table). Clinical and demographic information of patients are given in S1 Data. In the combined cohort, there were 212 (58.6%) male and 150 (41.4%) female patients. The male-to-female ratio (1.4:1) was consistent with published retrospective and prospective cohorts [20, 21]. The most common type of CDH was left-sided Bochdalek; rare forms of CDH or atypical lesion sides were also included (Table 1).

**Table 1:** Clinical summary of patients.

	Number	Percent
Gender		
Male	212	58.6%
Female	150	41.4%
CDH classification		
Isolated	208	57.5%
Complex	149	41.2%
Unknown	5	1.4%
Lesion side		
Left	270	74.6%
Right	56	15.5%
Eventration/Morgagni/Agenesis	11	3.0%
Unknown	25	6.9%
CDH type		
Bochdaleck	294	81.2%
Other^#^	22	6.1%
Unknown	46	12.7%
DHREAMS cohort (n = 283): Time of recruitment		
Neonatal	229	80.9%
Fetal	9	3.2%
Child	45	15.9%
Discharge vital status (n = 283)		
Survived	241	85.2%
Deceased	42	14.8%
Development assessment^¶^ (n = 283)		
At 2 years follow-up	152	53.7%
At 5 years follow-up	70	24.7%
No assessment at either 2 or 5 years	128	45.2%
Additional anomalies in complex cases (n = 149)		
Cardiovascular	66	44.3%
Neurodevelopmental^§^	37	24.8%
Skeletal	26	17.4%
Genitourinary	14	9.4%
Gastrointestinal	13	8.7%

¶ Development assessment at 2 years follow-up include Vineland Adaptive Behavior Assessment (Vineland-II) and/or Bayley Scales of Toddler Development (Bayley-III); tests at 5 years follow-up include Vineland-II and/or Wechsler Preschool and Primary Scale of Intelligence (WPPSI).

§Neurodevelopmental conditions include congenital abnormalities in central nerves system, and developmental delay or neuropsychiatric disorders based on the follow-up developmental evaluations.

A total 149 (41.2%) cases had additional congenital anomalies or neurodevelopmental disorders (NDD) at the time of last follow up and were classified as complex cases; and 209 (57.7%) patients had no additional anomalies at last contact were classified as isolated cases. The most frequent comorbidity among complex cases was cardiovascular anomalies (44.3%). NDD, skeletal malformations, and genitourinary defects were also observed in complex cases (Table 1).

### Burden of *de novo* coding variants

We identified 471 coding *de novo* variants in 264 (72.9%) cases including 430 single nucleotide variants (SNV) and 41 indels. Transition-to-transversion ratio of *de novo* SNVs was 2.64. The number of *de novo* coding variants per proband closely followed a Poisson distribution, with an average of 1.32 in WGS trios and 1.28 in combined WES trios (S2 Fig). Variants that were likely gene disrupting (LGD) or predicted deleterious missense (“D-mis” defined by CADD score [22] ≥25) were considered as damaging. A total of 193 damaging variants (57 LGD and 138 D-mis) were identified in 150 (41.4%) cases, including 38 (10.5%) cases harboring two or more such variants. Compared with the baseline expectations (Material and methods) [23], both *de novo* LGD variants (0.16 per case) and D-mis variants (0.38 per case) were significantly enriched in cases (fold enrichment (FE) = 1.73, P = 8.6x10^-5^ by one-sided Poisson test for LGD; FE = 1.5, P = 1.1x10^-6^ for D-mis) while the frequency of silent variants closely matched the expectation (0.30 per case, FE = 1.01, P = 0.48 by one-sided Poisson test).

Consistent with the previous study [16], damaging variants showed a higher enrichment in complex cases than isolated cases (FE = 1.70 vs 1.64 for LGD, 1.61 vs 1.38 for D-mis; S2 Table); and the proportion of complex cases who carried damaging variants was higher than isolated cases (43.6% vs. 39.4%). Burden of damaging variants was also higher in female than male cases (FE = 2.09 vs 1.47 for LGD, 1.63 vs 1.36 for D-mis; S2 Table), supporting a “female protective model” similar to autism and other NDD with male bias [24, 25].

Recent studies highlighting the use of large population reference sequencing data in interpreting LGD variants has demonstrated that genes depleted of LGD variants in the general population were more likely associated with disorders with reduced reproductive fitness[26]. We defined constrained genes by the estimated probability of loss-of-function intolerance (pLI) [27] ≥0.5 and found the burden of LGD variants was largely explained constrained genes (Table 2). D-mis also showed a higher enrichment in constrained genes (Table 2).

**Table 2:** Burden of *de novo* coding variants.

Gene Sets	Variant class	Number of variants	Baseline expectation	Fold enrichment	P-value
All Genes	Synonymous	110	109.1	1.01	0.48
	Missense	295	250.6	1.18	3.42E-03
	D-mis	138	93.7	**1.47**	**1.08E-05**
	LGD	57	32.9	**1.73**	**8.60E-05**
Constrained Genes	Synonymous	34	38.8	0.88	0.80
	Missense	112	88.1	1.27	7.91E-03
	D-mis	59	38.0	**1.55**	**9.39E-04**
	LGD	30	12.0	**2.50**	**9.05E-06**
Other Genes	Synonymous	76	70.3	1.08	0.26
	Missense	184	162.6	1.13	0.053
	D-mis	80	55.7	1.44	1.28E-03
	LGD	27	20.9	1.29	0.11

Constrained genes are defined by pLI metrics ≥0.5. LGD: likely gene disrupting, including frameshift, stop-gain, stop-loss, and variants at canonical splice sites; D-mis: predicted deleterious missense variants defined by CADD Phred score ≥25. The baseline expectations for different types of variants were calculated by the previous published method[23, 28]. The enrichment of observed number of variants was evaluated by a one-sided Poisson test.

### *MYRF* is a new syndromic CDH gene

We identified eight genes affected by more than one *de novo* LGD or missense variant (S3 Table). The top ranked gene, *MYRF*, has one frameshift insertion and three damaging missense variants, all of which were validated by Sanger sequencing. It is the only constrained gene in the list. By comparing with baseline expectations, only *MYRF* reaches genome-wide significance after Bonferroni correction of ~20000 coding genes (P = 5.3x10^-8^ <0.01/20000, by one-sided Poisson test).

Notably, all four patients with *MYRF* variants also had congenital heart disease (CHD), and three of them had genital anomalies including blind-ending vagina in a female and ambiguous genitalia or undescended testes in two male cases (Table 3). By screening another 220 CDH trios collected by the DHREAMS study, we identified another patient harboring a *de novo* splice acceptor site variant. The female patient had a diagnosis of Scimitar syndrome (a complex form CHD). She also had a monozygotic twin sister with hypoplastic left heart syndrome who also carried the same variant but no known CDH.

**Table 3:** Phenotype characteristics of patients with *de novo* coding variants in *MYRF*.

Study	Sample ID	Genetic Sex^#^	*De novo* variant (NM_001127392.2)	CADD Phred	Diaphragm defect	Cardiovascular defect	Urogenital defect	Other malformations
Current study	01–1008	XY	c.235dupG: p.G81Wfs*45	-	L-CDH	ASD,VSD,ToF	Bilateral undescended testes	No
	01–0429	XX	c.1303G>A:p.G435R	32	L-CDH	VSD	No internal genital organs, blind-ending vagina	Accessory spleen
	04–0042	XY	c.2036T>C:p.V679A	25.9	L-CDH	ASD,VSD	Unknown	Unknown (Deceased)
	05–0050	XY	c.2084G>A:p.R695H	34	CDH	HLHS	Ambiguous genitalia, undescended testes	Intellectual disability and motor delay at 2 years old
	01–0033	XX	c.1904-1G>A	25	R-CDH	Scimitar syndrome	Unknown	Unknown (Deceased)
	01–0591*	XX			No	HLHS	Unknown	Unknown (Deceased)
	CHU-11	XY	c.1786C>T:p.Q596*	37	No	Dextrocardia	Swyer syndrome with female genitalia	Right pulmonary hypoplasia
PCGC[29]	1–02264	XY	c.1160T>C:p.F387S	27.9	No	AAH, CoA, HLHS	Ambiguous genitalia, hypospadias, undescended testis	No
	1–03160	XY	c.1209G>C:p.Q403H	27.6	Right hemi-diaphragm eventration	Scimitar syndrome, AAH, ASD, BAV, HLHS, MS, VSD	Undescended testis	Lung hypoplasia
	1–07403	XY	c.1435C>G:p.L479V	23.9	No	BAV, CoA	Swyer syndrome with female genitalia	Short stature
Pinz et al.[30]	Case 1	XY	c.2336+1G>A	26.8	No	Scimitar syndrome, cor triatriatum	Penoscrotal hypospadias, micropenis, unilateral cryptorchidism	Mild speech delay, pulmonary hypoplasia, tracheal anomalies
	Case 2	XY	c.2518C>T:p.R840*	44	R-CDH	Scimitar syndrome	Persistent urachus, Undescended testis	Cleft spleen, thymic involution, thyroid fibrosis
Chitayat et al. [31]	Fetus case	XY	c.1254_1255dupGA: p.T419RfsX14	-	No	HLHS	Ambiguous external genitalia, right hepato-testicular fusion and left spleno-testicular fusion	Mild pulmonary hypoplasia, intestinal malrotation

Abbreviations: L/R-CDH, (lef/right)-congenital diaphragmatic hernia; AAH, aortic arch hypoplasia; ASD, atrial septal defect; BAV, bicommissural aortic valve; CoA, coarctation of aorta; VSD, ventricular septal defects; ToF, Tetralogy of Fallot; MS, mitral stenosis; PCGC, Pediatric Cardiovascular Genetics Consortium.

* 01–0591 is the monozygotic twin of 01–0033.

# Genetic sex is based upon the chromosome complement.

Given the strong association of *MYRF* variants with CHD, we then searched for *de novo* variants from a recently published study of CHD conducted by Pediatric Cardiac Genomics Consortium (PCGC) [29] and identified three additional *de novo* missense variants in *MYRF* from 2645 trios. All CHD patients also had genitourinary anomalies, including a patient with Swyer syndrome (46XY karyotype with female reproductive organs). One CHD patient with the Q403H variant had hemidiaphragm eventration. Recently, Pinz *et al*. [30] and Chitayat et al [31] reported three additional cases with complex CHD who carried *de novo* LGD variants in *MYRF*. All cases had genital defects, and one had CDH and the other two had pulmonary hypoplasia. Furthermore, from clinical WES, we also identified a Swyer syndrome patient with a stop-gain variant in *MYRF* who had dextrocardia and pulmonary hypoplasia.

**Fig 1:**
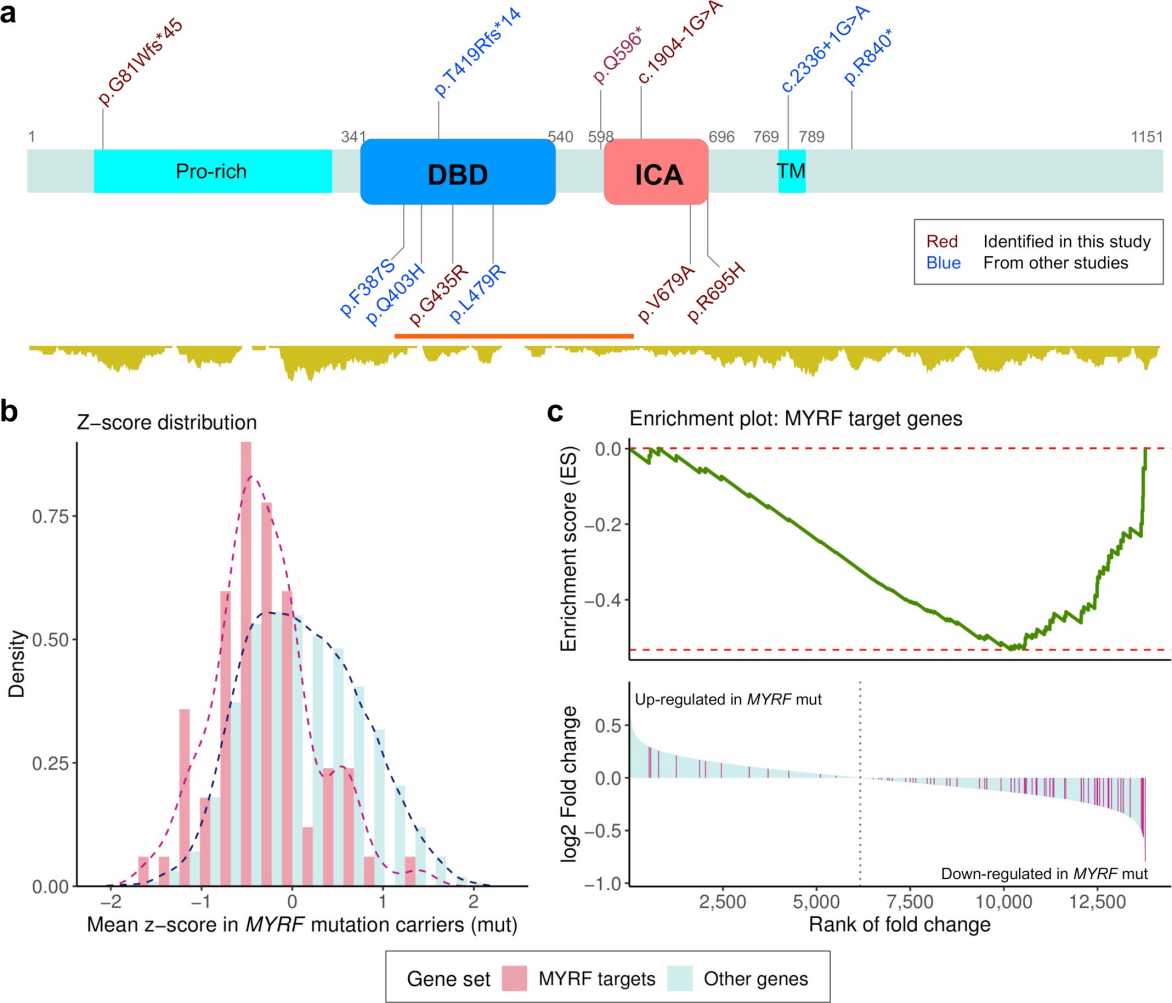
*De novo* coding variants in MYRF and their functional impact on transcriptome. (a) Schematic diagram of the MYRF protein structure. DBD: DNA binding domain; ICA: Intramolecular chaperone auto-processing domain; Pro-rich: proline-rich region; TM: transmembrane helix. The position of DBD and ICA were based on the annotation from InterPro, and Pro-Rich and TM were from SwissProt. The coordinates are given with respect to the canonical isoform (1151 amino acids). The relative position of 12 *de novo* coding variants are displayed, including 6 discovered in the current study (shown in red), and five from published studies of congenital heart disease (CHD) [29, 30] (shown in blue). LGD variants are shown on top of the protein; and missense variants are on the other side. Shown below the protein structure is the density of missense variants in gnomAD (http://gnomad.broadinstitute.org/). A missense constraint region [37] is highlighted in red (observed/expected number of missense variants = 0.31) (b) Z-score for each gene is the standardized expression level across samples. Mean Z-scores of MYRF target genes in three *MYRF* variant carriers were shifted to the lower end as compared with other genes. (c) Gene-set enrichment analysis (GESA) was applied to genes ranked by the estimated fold change of expression level comparing *MYRF* variant carriers with other cases. The MYRF target genes tend to have lower ranks and majority of them were down-regulated in *MYRF* variant carriers (NES = -2.10, P<5.0E-4).

In total, we identified 13 patients harboring 12 different *de novo* functional variants in *MYRF* (6 LGD and 6 missense variants; Fig 1A). All patients had CHD; and excluding those who died in infancy and had incomplete phenotypic information, all patients also had genitourinary anomalies. CDH was present in 7 out of 12 patients, and diaphragm defects were not systematically evaluated in cases without reported CDH. There was no clear phenotypic difference between patients with LGD variants and those with missense variants (Table 3). Taken together, the unique association of CDH and similar non-diaphragm defects including CHD, Scimitar syndrome, genitourinal anomalies and sex reversal in 46XY patients with *de novo* variants in *MYRF* establish it as a new syndromic CDH gene.

*MYRF* is a highly constrained gene in the population (pLI = 1). By examining both public databases (ExAC and gnomAD) and our own cohort, we only identified two rare LGD variants that affect all functional isoforms, yet their functional consequences were not clear (S5 Table). We also searched for inherited variants in 362 CDH trios and 2645 CHD trios from PCGC but did not find any inherited LGD variants in probands. Enrichment for *de novo* LGD variants associated with CDH and near complete absence of loss-of-function variants in the general population suggest that variants causing loss of *MYRF* function are likely fully penetrant for one or more aspects of this syndrome. All six *de novo* missense variants identified patients were also absent from the public databases, consistent with their high penetrance as LGD variants in this gene.

### Functional analysis of *MYRF* variants

*MYRF* is a membrane-associated transcription factor that plays a pivotal role in oligodendrocyte differentiation and myelination [32, 33]. Although it has not previously been implicated in diaphragm or cardiac development, its expression level was ranked at the top 21% of genes expressed in mouse developing diaphragm at E11.5 [34] and top 14% in developing heart at E14.5 [35].

The MYRF protein has two functional isoforms. Both isoforms contain a N-terminal proline-rich region followed by a DNA binding domain (DBD), which can be cleaved from the membrane by a region called intramolecular chaperon auto-processing (ICA) domain. All frameshift and stop gained variants resulted in truncated protein products in both functional isoforms and may trigger non-sense mediated decay. The precise functional effects of splice site variants were not evaluated, but are predicted to cause exon skipping, intron retention or activation of cryptic splice site and also result in a truncated protein. All six missense variants aggregated in the two DBD and ICA functional domains (Fig 1a). The missense variants were predicted as deleterious by a majority of bioinformatics tools (S4 Table). Most of the affected amino acid residues are highly conserved across species (S3 Fig).

MYRF DBD is homologous to yeast transcriptional factor Ndt80 but MYRF can only function as a trimer [36]. All missense variants in this domain are located in a region depleted of missense variants in the population (observed/expected = 0.31; Fig 1A) and have high MPC scores [37] (S4 Table). Protein structure modeling predicted that those variants may affect DNA binding affinity (F387S), change surface charge distribution (Q403H), or destabilize the protein structure (G435R and L479R) (S4 Fig).

Previous studies also showed that full length MYRF forms a trimer before cleavage, and trimerization is required for auto-cleavage and subsequent activation [38]. The ICA domain which is distantly related to bacteriophage’s tailspike protein was believed to play an essential role in MYRF trimerization. Two missense variants (V679A, R695H) are located at the C-terminal end of the ICA domain where the triplet helix bundle is formed [39]. V679 is one of the critical residues in ICA that is fully conserved from human to bacteriophage (S3 Fig). Structure modeling predicted that the variant R695H may destabilize the trimer structure (S4 Fig) and would fail to produce functional MYRF DBD trimers by trimerization-dependent auto-proteolysis.

To evaluate the effect of *MYRF* variants on gene expression, we performed RNA-seq on diaphragm fibroblast cell cultures from neonatal patients. After removing outlier samples (S5 Fig), we obtained transcriptome data of 31 patients including three with a *de novo MYRF* variant (one frameshift insertion and two missense variants in the ICA domain). Most patients (27/31, 87%) included in the RNA-seq analysis were self-reported non-Hispanic White. Additionally, we identified 74 putative MYRF target genes from a previous study of rat oligodendrocyte progenitor cells (S3 Data) [40]. Gene expression levels were quantified as TPM (transcripts per million mapped reads). The z-scores of expression levels of putative MYRF target genes were systematically shifted down in *MYRF* mutant cells (P = 2.4E-7 by Kolmogorov-Smirnov test; Fig 1B), consistent with the reduced transcription factor activities caused by the damaging variants. We quantified differential expression (DE) of genes between samples with and without *de novo MYRF* variants by a shrinkage estimator of fold change [41]. Selected DE genes were validated by quantitative polymerase chain reaction (qPCR) on the same cell cultures (S7 Fig). Using gene set enrichment analysis [42] of genes ranked by the fold changes, putative MYRF target genes are significantly enriched among the down-regulated genes (normalized enrichment score (NES) = -2.10, P<5.0E-4; Fig 1C). Since all *MYRF* mutation carriers were males, we repeated the analysis using only males and found the results are similar as using all samples (NES = -1.95, P<5.0E-4), suggesting that sex is not a confounding factor. The patient with the *MYRF* frameshift variant was the only *MYRF* mutation carriers whose ethnicity was not self-reported White. The enrichment of MYRF target genes is also observed in genes down-regulated in the two samples with missense variants (S6 Fig), suggesting that the result was not driven by the LGD variant or ethnicity.

Manual inspection of top DE genes (S4 Data) revealed that *GATA4*, a known CHD gene that has also been implicated in familial and sporadic CDH [43], was significantly down-regulated in cases with *de novo MYRF* variants (estimated fold change = 0.54, q-value = 0.03). Interestingly, we observed that expression trajectories of *MYRF* and *GATA4* were similar in mouse developing diaphragm and lung (S8 Fig) suggesting that they play similar functional roles during diaphragm and pulmonary development.

Besides *MYRF*, we estimated there were 64 (95% CI: 38–93) genes with *de novo* variants implicated in CDH based on the overall burden analysis. Most of those genes have only one damaging variant in the cohort. To prioritize among all the genes with *de novo* damaging variants, we took two approaches.

### Genetic overlap with other disorders

We noted that CHD was the most common non-diaphragm defect in complex cases (Table 1). Damaging mutations in *MYRF* have been identified in a previous CHD study but the gene did not reach genome-wide significance [29]. The identification of the *MYRF* syndrome suggested that the comorbidity of CHD and CDH in some cases can be explained by the same genetic factors, many of which remain to be discovered. CDH is also part of the phenotype spectrum of several rare Mendelian disorders [8]. Recently discovered genes for developmental disorders are often pleiotropic and implicated in multiple diseases [15, 29, 44]. Thus, the finding of *MYRF* motivated us to assess the genetic overlap between CDH and other developmental disorders, especially CHD, to help us prioritize additional CDH genes with pleiotropic effects. To this end, we curated genes that were known or implicated in CHD and other developmental disorders (S5 Data; Materials and Methods). Hereafter we refer to these known or candidate genes as CHD or DD genes.

**Fig 2:**
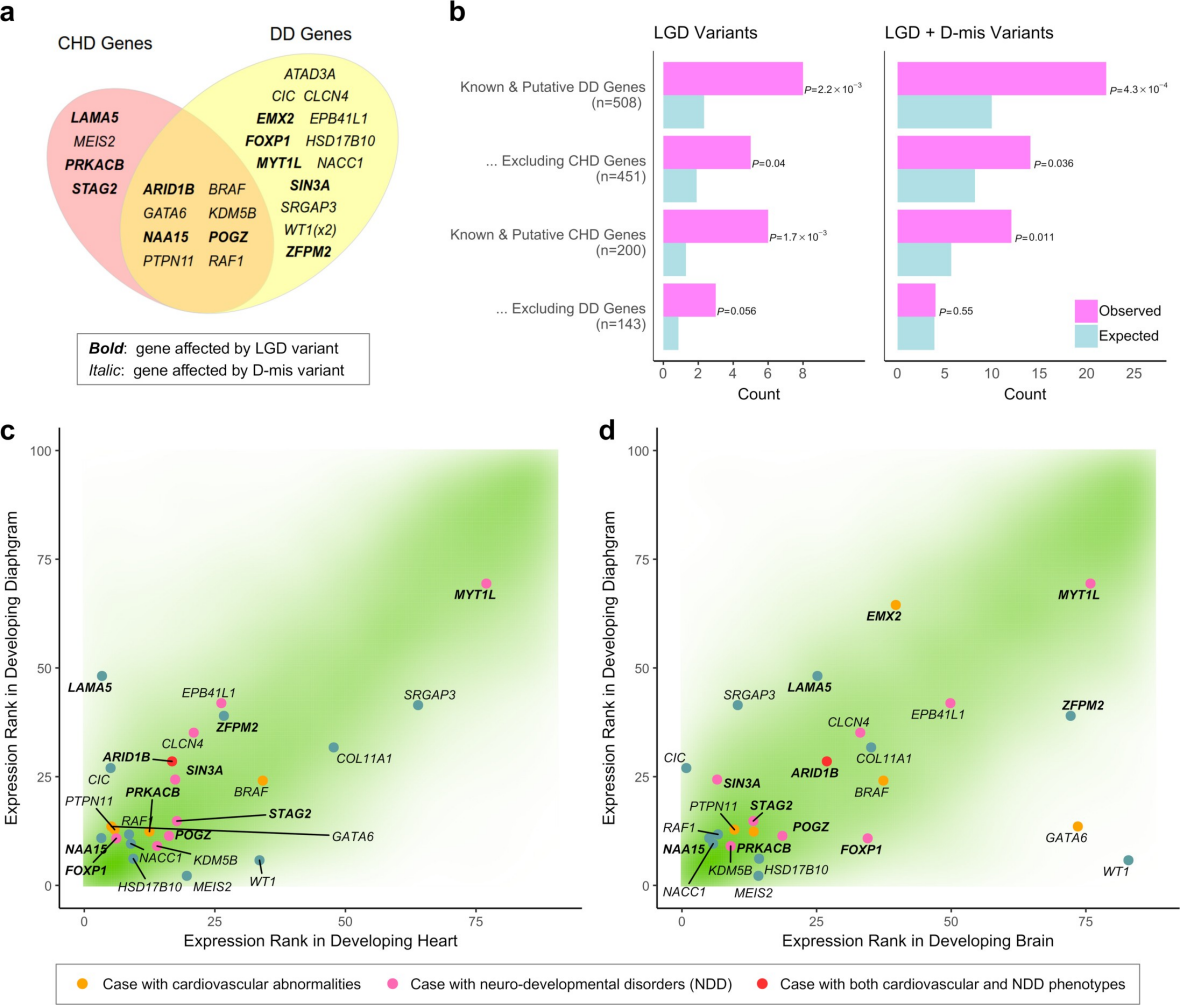
Genetic overlap with other developmental disorders. (a) Venn diagram shows 25 genes implicated in developmental disorders and congenital heart disease (DD and CHD genes, Materials and Methods) that are affected by damaging variants in CDH. (b) Enrichment of LGD and D-mis variants in DD and CHD genes. Enrichment was evaluated by comparing the observed number of *de novo* damaging variants in DD and CHD genes with the expected number of hits by randomly scattering the same number of variants to the exome while controlling for the number of variants, gene length, and sequence context (Materials and Methods). (c) Expression percentile ranks in the developing diaphragm [34], heart [70] and brain [70] are shown for all genes (green density), highlighting DD and CHD genes listed in (b). Smaller ranks correspond to higher expression levels.

In addition to *MYRF*, we identified a total of 26 DD/CHD genes with damaging *de novo* variants in 25 CDH patients (Fig 2A). Using a simulation approach that accounted for the number of variants, gene size, and sequence context (Materials and Methods), we found that damaging variants in CDH were significantly enriched in the DD and CHD genes (Fig 2B). For example, we observed 6 CHD genes with *de novo* LGD variants in CDH which was 4.7-fold higher than expected (P = 1.7x10^-3^); the number of DD genes with *de novo* LGD variants (8) was 3.4 folder higher than expected (P = 2.3x10^-3^). Among CHD genes with at least one damaging variant in CDH, haploinsufficiency of *WT1* is a known cause of several syndromic forms of CDH [8]; *ZFPM2* and *GATA6* have already been established as CDH genes by previous studies [45, 46]. However, the enrichment of damaging variants and especially LGD variants remained significant after excluding known or candidate CDH genes [47] (S9 Fig). Furthermore, the enrichment cannot fully be explained by the over-representation of constrained genes, because the enrichment persisted after conditioning on all constrained genes and remained significant for LGD variants (S9 Fig).

The cross-disease overlap suggests that pleiotropic effects of variants in the genes associated with other developmental disorders are also associated with CDH in a fraction of cases. Since CHD genes were curated based on the damaging mutations in CHD patients and DD genes were mostly implicated in other developmental disorders, the genes that appear in both sets were more likely to participate in a broader range of developmental process. Accordingly, the enrichment in genes found exclusively in one set was significantly reduced (Fig 2B, S9 Fig).

We reviewed the most recent medical records of those patients (S7 Table) and identified six complex cases with CHD and/or NDD compatible with the initial reported phenotypes for these genes. Two additional cases were found to have non-CHD cardiovascular defects like two-vessel cord or dilated aortic root; and another four had mild-to-moderate developmental delay/intellectual disability at latest evaluation. Four patients who carried LGD variants in known DD genes (*POGZ*, *ARID1B*, *FOXP1*, and *SIN3A*) and one patient who carried a known activating variant in the Noonan syndrome gene *PTPN11* were considered pathogenic variants by the American College of Medical Genetics and Genomics guidelines [48].

**Fig 3:**
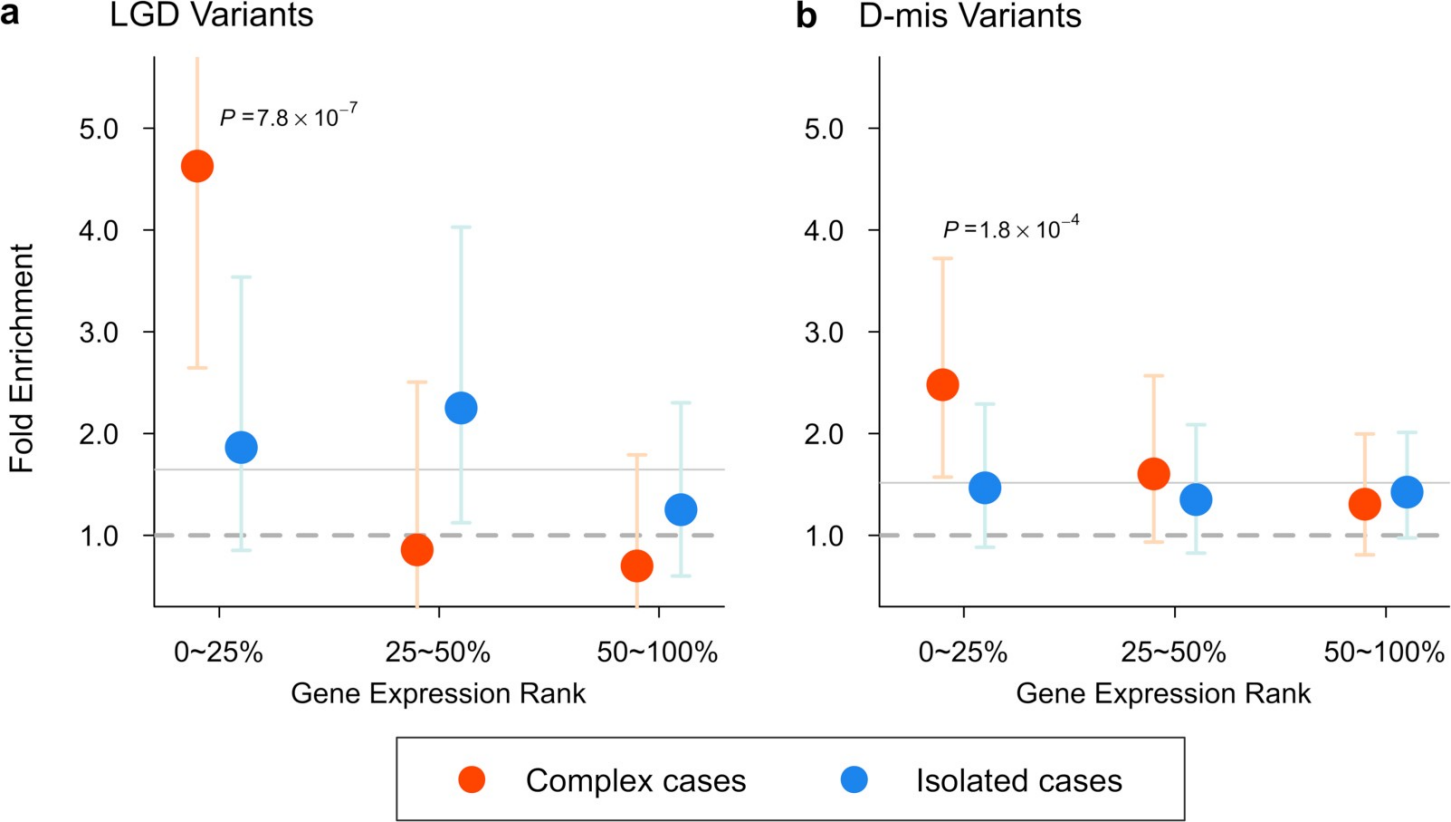
Burden of damaging *de novo* variants in different gene sets and sub classes of CDH. (a) In complex cases, LGD variants were dramatically (4.6 fold) enriched in genes highly expressed (ranked in the top quartile) in mouse developing diaphragm (MDD) [34], and showed no enrichment in other quartiles. By comparison in isolated cases, LGD variants showed similar enrichment (~2 fold) across expression levels. (b) D-mis variants carried by complex cases also showed highest (2.4 fold) enrichment in the top quartile of MDD expression. Enrichment was evaluated by comparing observed number of variants to the baseline expectation[23, 70] using a one-sided Poisson test. Bars represent the 95% confidence intervals of estimated fold enrichment.

Pleiotropy was further supported by the gene expression data. The majority of the 26 DD/CHD genes with damaging *de novo* variants in CDH were not only highly expressed in mouse developing diaphragm but also in developing heart or brain (Fig 2C). Indeed, over all coding genes, expression ranks in the three developing organs were highly correlated (Spearman rank correlation r = 0.74 between diaphragm and heart, 0.74 between diaphragm and brain). Therefore, high diaphragm expression can be a proxy for a pleiotropic effect. Consistent with this, we found that all damaging *de novo* variants in complex cases, presumed to enrich causative variants affecting multiple organs, were greatly enriched in genes at the top quartile of expression in developing diaphragm (FE = 4.6, P = 7.9x10^-7^ by one-sided Poisson test for LGD; FE = 2.4, P = 1.8x10^-4^ for D-mis). By contrast, in isolated cases, the enrichment of damaging variants was distributed in genes across a broad range of expression (Fig 3).

### Functional enrichment map

**Fig 4:**
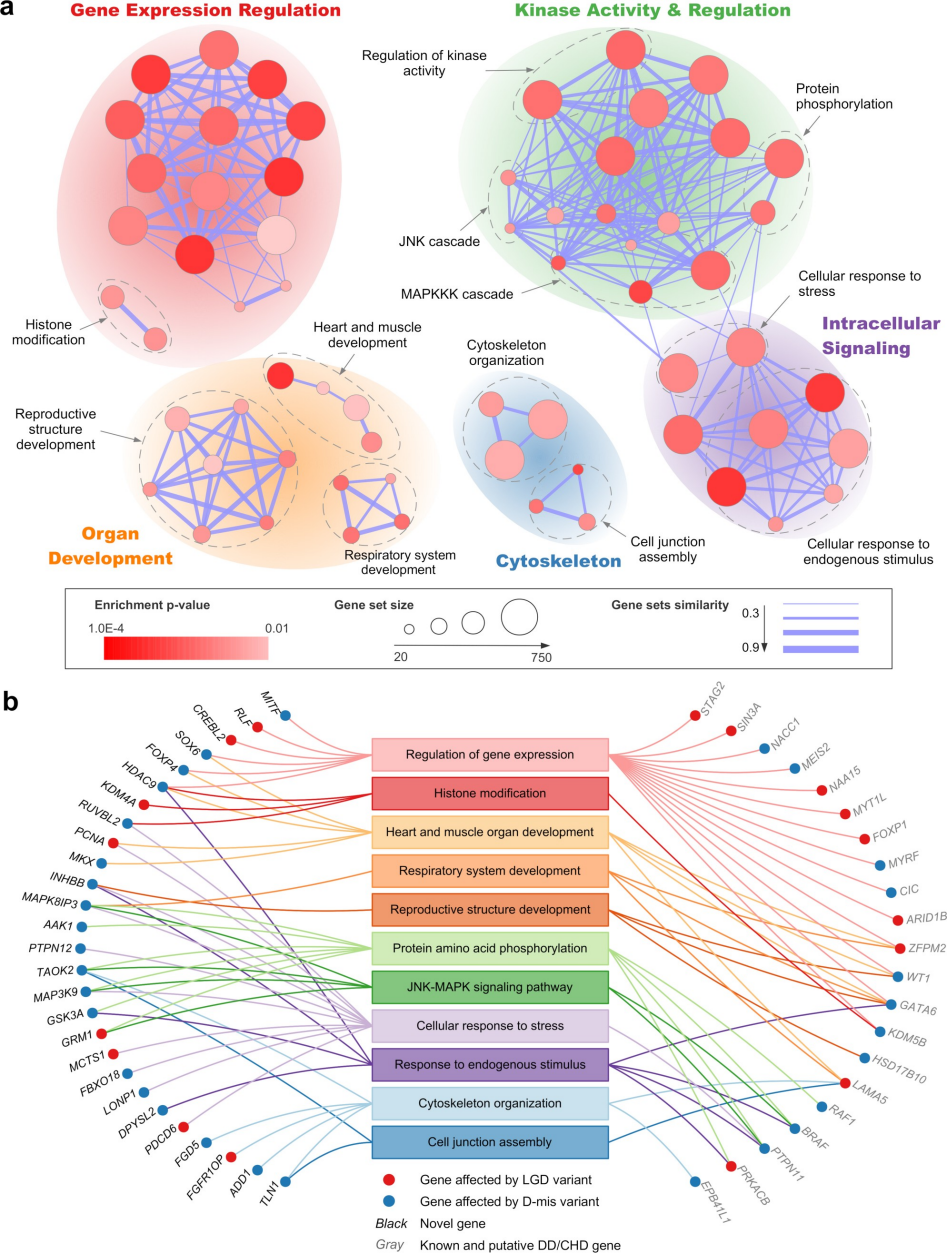
A functional enrichment map of genes affected by *de novo* damaging variants in CDH. (a) Enrichment results were visualized by a network of gene sets, where node size is proportional to the number of genes in each gene set and the thickness of edge represents the overlaps between gene sets. The significance of enrichment (p-value) is indicated by the color gradient. Functionally related gene sets are circled and manually labeled. Sub-clusters of network with similar functional annotations are grouped together as functional modules. (b) Mapping genes affected by damaging variants in CDH to the enriched functional groups shown in (a).

As a second approach to prioritize CDH genes, we hypothesized that different CDH genes converge onto a small number of pathways, and novel genes in the enriched pathways could be candidates for new disease genes. We evaluated functional enrichment of genes affected by damaging *de novo* variants to identify biological processes involved in CDH. To boost the signal, only constrained genes or known haploinsufficient genes were included in the pathway analysis (Materials and Methods). A total of 63 Gene Ontology Biological Process gene sets were enriched at a false discovery rate (FDR) of 0.1 (S6 Data). To remove the redundancies between gene sets, we used a similarity score to organize functionally related gene sets into a network. The resulting network was annotated and visualized as a functional enrichment map (Fig 4A). Eleven functional groups were identified that recapitulated our current knowledge about the molecular genetic basis of CDH [49]. They were supported by 48 genes including 27 novel genes (Fig 4B).

Transcription factor haploinsufficiency is an established cause of CDH [50] and other birth defects [51]. Recently, disruption of epigenetic machinery was also found to underlie many developmental disorders [35, 44, 52]. The majority of DD/CHD genes directly or indirectly regulate gene expression which formed a highly connected cluster of enriched gene sets, some of the transcription factors are involved in the development of heart, lung and reproductive organs. We identified nine novel genes encoding transcription factors or histone modifiers.

Proper cell migration is critical during diaphragm development. Initially, mesenchymal precursor cells migrate from mesoderm to form the primordial diaphragm. After that, pleuroperitoneal folds of the primordial diaphragm become the targets of migration of muscle progenitors, where they undergo myogenesis and morphogenesis [53]. Several related pathways were implicated including cellular response to growth factors or stress events that initiate directional migration [54], actin cytoskeletal organization and cell-cell junction assembly that drive and fine tune cell movement [55, 56]. Gene sets in protein phosphorylation and JUN-MAPK (mitogen-activated protein kinase) cascades were also enriched but not entirely due to three Noonan syndrome genes (*PTPN11*, *BRAF*, *RAF1*). The enrichment in kinase activity related pathways was supported by six novel kinase genes that overlapped with intracellular signaling functions. One kinase gene, *MAPK8IP3*, has been implicated in lung development in a mouse model [57].

## Discussion

In this study, by analyzing *de novo* coding variants in CDH, we confirmed the overall enrichment of damaging *de novo* variants and identified *MYRF* as a new syndromic CDH gene. All our CDH cases with *MYRF* mutations also had CHD and most of them had genitourinary defects. The striking phenotypic similarities among the cases suggest that damaging *de novo* variants of *MYRF* disrupt the function of progenitor cells of developing diaphragm, heart and reproductive organs. In this novel *MYRF* syndrome, all cases with disease associated variants had CHD including three with Scimitar syndrome, whereas penetrance CDH was incomplete. It suggests that the manifestation of CDH in this syndrome depends on other genetic, environmental, or stochastic factors. The monozygotic twin case discordant for CDH supports that stochastic developmental events are involved.

*MYRF* is well known for its function in regulating myelination of the central nervous system [32]. A mouse model with conditional deletion of *MYRF* in oligodendrocyte precursors has abnormal motor skill [58]. Recently, an inherited missense variant in *MYRF* (Q403R) has been reported as the cause of encephalopathy with reversible myelin vacuolization in a Japanese pedigree [59]. This variant is located at the same residue as the *de novo* missense variant in one of the PCGC cases but with a different substutition (Q403H). No other congenital defects were reported for the variant carriers in that family. The Q403R variant has been experimentally shown to diminish the transcription activity of a target gene [59], similar to our finding in two other missense variants (S6 Fig). Why the two different substitutions at the same amino acid position result in different phenotypes remains to be elucidated in the future. Among patients with *de novo* damaging variants in *MYRF*, one individual with the R695H variant also had intellectual disability and delayed motor skills (Table 3).

We identified 25 other individuals harboring damaging *de novo* variants in known or candidate DD/CHD genes, most of which have not been reported to be associated with CDH before. The significant enrichment of damaging variants among DD/CHD genes strongly suggest their causative role for majority of these cases. Similar to the case of *MYRF*, many DD/CHD genes have yet to be established as known disease genes. The enrichment of CDH damaging variants support their possible involvement in a broader range of developmental abnormalities which should be further evaluated in additional case cohorts with other congenital anomalies. Some recent studies of other congenital anomalies and developmental disorders have already provided further evidence for a few putative DD/CHD genes. For example, a damaging missense variant in *LAMA5*, a gene that plays a role in the maintenance and function of the extracellular matrix critical for pattern formation during development [60], was associated with multi-system syndrome in an Italian family [61]. Duplication of *STAG2*, which encodes a subunit of cohesin complex, was associated with intellectual disability and behavioral problems [62]. *MEIS2* was previously nominated as a potential CDH candidate by transcriptome analysis [34] and encodes an interaction partner of transcription factor gene *PBX1*, haploinsufficieny of which has recently been associated with multiple developmental defects including CDH [63].

Since our knowledge of DD/CHD genes is incomplete, it is possible that this observed genetic overlap represents only the tip of an iceberg. Our pathway analysis not only captured general biological process during developmental, but also identified pathways that are closely related to diaphragm development. Some novel genes prioritized by the pathway analysis have also been supported by new genetic data in other disorders. For example, *de novo* copy number loss or missense variants in *TAOK2*, one of the kinase gene implicated by the enriched gene sets of the kinase activity and MAPK signaling, has been demonstrated to cause autism and other NDD [64]. Because CDH is a relatively uncommon and lethal condition as are many other rare congenital anomalies, it is difficult to recruit large numbers of patients for genetic studies. The findings from this and other studies [15] suggest that cross-disorder analysis can be a powerful strategy for future gene discovery.

The genetic overlap between CDH and other disorders is consistent with pleiotropy among developmental disorder genes and is further supported by the highly correlated gene expression levels in multiple developing organs. We also showed that different enrichment patterns of *de novo* damaging variants between complex and isolated CDH cases is consistent with the hypothesis that variants in complex cases affect genes with more pleiotropic effects.

The pleiotropic effects of genes during development also suggest that our current classification of “isolated” cases may understate their non-diaphram abnormalities. A limitation of our study is the lack of long term clinical outcome data on many of the patients since our cohort is still relatively young. Examining the most recent medical records of patients with variants in DD/CHD genes revealed mild-to-moderate cadiovascular or NDD symptoms in several cases initially classified as isolated at birth (S7 Table). The medical records were often incomplete for patients who died at early infancy or were lost to follow-up (Table 1), and it is likely that NDD outcome in many isolated patients were underestimated [65, 66]. Furthermore, almost all isolated cases also had pulmonary hypoplasia. Traditionally it was assumed that lung defects were caused by the mechanical compression by the herniated visceral, but it is clear now that development of lung and diaphargm are two intricatelly connected developmental processes [67], and lung defects may share common etiologies with CDH [68]. Among *MYRF* variant carriers, four patients who did not have diaphragm defects developed pulmonary hypoplasia (Table 3), further supporting common genetic control of these two processes. Larger cohorts with more detailed neurodevelopmental and long term outcomes will enhance our ability to identify additional CDH genes and provide accurate prognostic information to families to allow for future clinical diagnosis of these conditions.

In summary, our analysis of *de novo* coding variants in 362 CDH trios identified a new disease gene *MYRF*, revealed genetic overlap with other developmental disorders, and identified biological processes important for diaphragm development. Future studies will beneifit from larger sample sizes, analyzing different types of genetic variants, leveraging the information from other developmental disorders, and integrating functional genomic data.

## Materials and methods

### Patients recruitment

Study subjects were enrolled by the DHREAMS study (http://www.cdhgenetics.com/). Neonates, children and fetal cases with a diagnosis of diaphragm defects were eligible for DHREAMS. Clinical data were collected from the medical records by study personnel at each of 16 clinical sites. A complete family history of diaphragm defects and major malformations was collected on all patients by a genetic counsellor. A blood, saliva, and/or skin/diaphragm tissue sample was collected from the patient and both parents. All studies were approved by local institutional review boards, and all participants or their parents provided signed informed consent.

Cases without known pathogenic chromosome abnormalities or copy number variations [11] were selected for exome or whole-genome sequencing. A total of 283 trios with no family history of CDH with three generation and not born to consanguineous marriages were included in the current study. *De novo* coding variants on a subset trios (n = 39) have been described in our previous study [17]. In Neonates cohort, longitudinal follow-up data including Bayley III and Vineland II developmental assessments since discharge at 2 years and/or 5 years of age were gathered. Patients were evaluated to have developmental delay if at least one of the composite scores was 2 standard deviations below population average.

Patients with additional birth defects or developmental delay or other neuropsychatric phenotypes at last contact were classified as complex, and otherwise as isolated. Pulmonary hypoplasia, cardiac displacement and intestinal herniation were considered to be part of the diaphragm defect sequence and were not considered to be additional birth defects.

Subjects of BCH/MGH cohort were enrolled in “Gene Mutation and Rescue in Human Diaphragmatic Hernia” study as described previously [16]. Among 87 trios from BCH/MGH cohort, 8 trios were found to be duplicates with DHREAMS trios and were excluded from the analysis.

### Whole exome/genome sequencing

Exome sequencing was performed in 79 trios that were not published before. Eleven trios were processed at the New York Genome Center. The DNA libraries were prepared using the Illumina TruSeq Sample Prep Kit (Illumina). The coding exons were captured using Agilent SureSelect Human All Exon Kit v2 (Agilent Technologies). Samples were multiplexed and sequenced with paired-end 75bp reads on Illumina HiSeq 2500 platform according to the manufacturer’s instructions. Sixty-eight trios processed at University of Washington Northwest Genome Center were captured using NimbleGen SeqCap EZ Human Exome V2 kit (Roche NimbleGen), and sequeced on HiSeq 4000 in 75 bp paired-end reads.

Another 192 trios were processed at Baylor College of Medicine Human Genome Sequencing Center using whole genome sequencing as part of the Gabriella Miller Kids First Pediatric Research Program. Among these, 27 trios were included in the previous exome study [17] but had no damaging *de novo* variants. Genomic libraries were prepared by the Illumina TruSeq DNA PCR-Free Library Prep Kit (Illumina) with average fragment length about 350 bp, and sequenced as paired-end reads of 150-bp on Illumina HiSeq X platform.

### *De novo* variant calling and annotation

Exome and whole-genome sequencing data were processed using an inhouse pipeline implementing GATK Best Practice (version 3). Briefly, reads were mapped to human genome reference (GRCh37) using BWA-mem (version 0.7.10); duplicated reads were marked using Picard (version 1.67); variants were called using GATK (version 3.3–0) HaplotypCaller to generate gVCF files for joint genotyping. All samples within the same batch were jointly genotyped and variant quality score recalibration (VQSR) was performed using GATK. Common SNP genotypes within exome regions were used to valid parent-offspring relationships using KING (version 2.0) [69].

A variant that was presented in the offspring and had homozygous reference genotypes in both parents was considered to be a potential *de novo* variant. We used a series of stringent filters to identify *de novo* variants as described previously[70]. Briefly, we first kept variants that passed VQSR filter (tranche≤99.8 for SNVs and ≤99.0 for indels) and had GATK’s Fisher Strand≤25, quality by depth≥2. Then we required the candidate *de novo* variants in proband to have ≥5 reads supporting alternative allele, ≥20% alternative allele fraction, Phread-scaled genotype likelihood ≥60 (GQ), and population allele frequency ≤0.1% in ExAC; and required both parents to have > = 10 reference reads, <5% alternative allele fraction, and GQ≥30.

We used ANNOVAR [71] to annotate functional consequence of *de novo* variants on GENCODE (v19) protein coding genes. All coding *de novo* variants were manually inspected in the Integrated Genomics Viewer (http://software.broadinstitute.org/software/igv). A total of 169 variants were selected for validation using Sanger sequencing; all of them were confirmed as *de novo* variant. The number of coding *de novo* variants per proband was compared with expectations under Possion distribution.

All coding variants were classified as silent, missense, inframe, and likely-gene-disrupting (LGD, which includes frameshift indels, canonical splice site, or nonsense variants). The most severe functional effect was assigned to each variant. We defined deleterious missense variants (D-mis) by phred-scaled CADD (version 1.3) [22] score≥25.

### *De novo* variant burden analysis

Baseline rate for different classes of *de novo* variants in each GENCODE coding gene were using a previously described mutation model [23, 70]. Briefly, the tri-nucleotide sequence context was used to determine the probability of each base in mutating to each other possible base (precomputed rates are available at: https://github.com/jeremymcrae/denovonear/blob/master/denovonear/data/rates.txt). Then, the mutation rate of each functional class of point mutations in gene was calculated by adding up point mutation rates in the longest transcript. The rate of frameshift indels was presumed to be 1.1 times the nonsense mutation rate. The expected number of variants in different gene sets were calculated by summing up the class-specific variant rate in each gene in the gene set mutiplied by twice the number of patients (and if the gene is located on the non-pseudoautosomal region of chromsome X, further adjusted for female-to-male ratio [14]). The observed number of variants in each gene set and case group was then compared with the baseline expectation using Poisson test.

In burden analysis, constrained genes were defined by pLI metrics [27] ≥0.5 which include a total of 5451 GENCODE genes, and all remaining genes were treated as other genes. We used a less stringent pLI threshold than previously suggested [27] for defining constrained genes, because it captured more known haploinsufficient genes important for heart and diaphragm development. Genes were also grouped by their expression levels in mouse developing diaphragm. Microarray expression profile of mouse pleuroperitoneal folds at E11.5 was taken from a previous study [34]. Normalized gene expression levels were converted to rank percentiles with smaller values corresponding to higher expression. Human orthologs of mouse genes were identified using annotations from MGI database (http://www.informatics.jax.org/). When a human gene mapped to multiple mouse genes, the highest expression level was assigned to the human gene.

### RNA sequencing

Fibroblasts were obtained from diaphragm biopies at the time of diaphragm repair from 36 CDH neonatal cases most of whom carried damaging *de novo* variants, including three cases carrying *MYRF* variants (p.G81Wfs*45, V679A, and R695H). Cells were cultured in Dulbecco's Modified Eagle's Medium supplemented with 10% heat-inactivated fetal bovine serum and 1x Antibiotic/antimycotic (Gibco; Life Technologies), following standard conditions. Cells were cultured in parallel in successive passes until optimal confluence was reached, and were collected with 2.5% Trypsin (Gibco; Life Technologies) and harvested by centrifugation 5 minutes at 1200rpm. Total RNA was extracted from the cell pellet of each subject using RNeasy LipidTissue mini Kit (QIAGEN) according to manufacturer's protocol. The quality and quantity of RNA were assayed using a Qubit RNA Assay Kit in a Qubit 2.0 Fluorometer (Life Technologies) and RNA Nano 6000 Assays on a Bioanalyzer 2100 system (Agilent Technologies). cDNA libraries were prepared with the TruSeq Stranded Total RNA Sample Preparation kit (Illumina), following the manufacturer instructions. And the purified products were evaluated with an Agilent Bioanalyzer (Agilent Technologies). The library was sequenced on Illumina HiSeq 2000 platform in 100-bp paired-end reads.

### RNA-seq data analysis

RNA-seq reads were mapped to the human reference genome (GRCh37) using STAR (version 2.5.2b) [72]. Gene expression levels were quantified as TPM from the output of FeatureCounts (2015–05 version) [73]. Only protein coding genes were kept for analysis and genes with no mapped reads in at least half of the samples were filter out. All sequenced samples had >20 million mapped read pairs with >90% mapping rate. Principle component (PC) analysis of gene expression profile showed that five samples were separated from others on the first two PC axes (S5 Fig). The outlier samples were likely due to different number of passages in cell culture, and were removed from analysis.

Differential expressed genes (DEG) between cases with *MYRF* variants and others were identified using DESeq2 package [41]. DEG were selected using following criteria: adjusted p-value < 0.5 and adjusted fold change > 0.5 or < -0.5. We noted that all three *MYRF de novo* variant carriers were male. To avoid confounding effect of gender, DEG analysis was also performed by comparing male samples with or without *MYRF* variants. The full DEG list is given in S4 Data.

To evaluate the consequence of *MYRF* damaging variants on patients’ transcriptome, we tested if putative MYRF target genes were systematically down-regulated in the fibroblast cells with *MYRF* variants using gene set enrichment analysis (GSEA). The MYRF target genes as oligodendrocyte-specific genes that had at least one MYRF ChIP-seq binding peaks with 100kb of transcription start site [40]. We then identified corresponding human orthologs using biomaRt package [74]. A total of 74 human genes were defined as putative target genes for GSEA.

### Quantitative PCR

We selected six genes from differentially expressed genes between *MYRF* mutation carriers and other cases, including four down-regulated (*GATA4*, *DBNDD2*, *MYO1D* and *NFASC*) and two up-regulated (*H3F3C* and *SEMA3A*) in *MYRF* mutant cells. First-strand cDNA was synthesized from the total RNA (500ng~1 µg) using the RNA to cDNA EcoDry Premix (Random Hexamers) kit (TaKaRa) according to manufacturer's instructions. Primers for the selected genes (S6 Table) were synthesized by IdtDNA. All qPCR reactions were performed in a total of 10 µl volume, comprising 5 µl 2x SYBR Green I Master Mix (Promega), 1 µl 10nM of each primer and 2 µl of 1:20 diluted cDNA in 96-well plates using CFX Connect Real-Time PCR Detection System (Bio-Rad). All reactions were performed in triplicate and the conditions were 5 minutes at 95°C, then 40 cycles of 95°C at 15 seconds and 60°C at 30 seconds. The relative expression levels were calculated using the standard curve method relative to the β-actin housekeeping gene. Five-serial 4-fold dilutions of cDNA samples were used to construct the standard curves for each primer.

### Cross-disorder genetic overlap

To assess the genetic overlap with other developmental disorders and especially CHD, we tested if the *de novo* damaging variants in CDH cases were enriched in known and putative CHD and DD genes. DD genes were extracted from DDG2P database [75] (accessed on Jan 11, 2018) and filtered to keep “allelic requirement” as monoallelic, X-linked dominant or hemizygous, and required “organ specificity list” to include brain, heart or not specific to any organ. A total 508 DD genes were identified, including 460 confirmed DD genes. CHD genes were collected based on a recent exome study of 2645 trios [29]. CHD genes included high heart expressed genes (HHE; ranked at top 25%) or known human CHD genes that were affected by more than one damaging de novo variants (LGD or D-mis defined by meta-SVM [76] as the original publication on CHD [29]) or constrained (pLI≥0.5) HHE genes affected by only one damaing variants from the same study. A total 200 CHD genes were identified, 57 of which overlapped with DD genes.

To assess if the exome-wide *de novo* damaging variants in CDH were enriched in CHD and DD genes, simulations were done to randomly place variants to the coding regions in a way that keeps the number of variants, tri-nucleotide context, functional effect, and deleteriouness prediction the same as that of the observed data [77]. Here the coding region was defined as coding sequences and canonical splice sites of all GENCODE v19 coding genes. For damaging mutations identified from WES data, the coding regions were restricted to the regions that have at >10X coverage in least 80% samples. Empirical p-value was calculated as the chance when there were more simulated damaging variants than observed in the given gene set. We ran 50,000 simulations to evaluate the significance. And the expected number of variants in a gene set was the average number of randomly generated variants in a gene set over all simulations.

### Functional enrichment map

To evaluate the functional convergence of genes affected by damaging variants, we extracted 89 genes that included 86 constrained genes (pLI≥0.5), two known candidates for CDH (*GATA6*, *WT1*), and a known haploinsufficient gene (*KDM5B*). Gene sets were derived from Gene Ontology Biological Process (GO-BP, accessed Feb 1^st^, 2018). The GO-BO categories that were statistically over-represented in the gene list (FDR<0.1) were identified using hyper-geometric test implemented by BINGO [78]. Terms annotating more than 750 or less than 25 genes were discarded, because large gene-sets usually represent broad categories without specific biological meaning. Small gene sets on the other hand are not likely to produce statistically significant results.

Enriched gene sets were graphically visualized as a network, in which each gene set is a node and edges represent overlap between sets. The Cytoscape software [79] and EnrichmentMap plugin [80] were used to construct the network. The color gradient of nodes reflects the enrichment p-values. Node size is proportional to the number of genes in the gene set. Edge thickness is proportional to the similarity score between gene sets which is defined by the average of Jaccard coefficient and overlap coefficient [80]. Enriched gene sets with highly overlapping genes (S6 Data) were grouped together and annotated manually.

## Supporting information

S1 Fig Depth of coverage. Scatter plot and marginal histograms for mean depth and percentages of targeted regions with at least 10 or 15 reads are shown for whole-exome sequencing (a) and whole-genome sequening samples (b).
(PDF)

S2 Fig Distribution of *de novo* coding variants per proband. Distribution of number of de novo coding variants per proband in whole-exome (a and b) and whole-genome sequenced trios (c).
(PDF)

S2 Fig Multiple sequence alignment of the DBD domain (a) and ICA domain (b) of the MYRF protein.
(PDF)

S4 Fig The predicted effects of *de novo* missense variants MYRF 3D structure. The predicated local 3D structures of wild and mutant type proteins are shown for F387S (a), Q403H (b), G435R (c), L479R (d), and R695H (e). V679A cannot be modeled due to lack of homologues template.
(PDF)

S5 Fig Principle component analysis of RNA-seq samples before (a) and after (b) removing outliers.
(PDF)

S6 Fig (Related to Fig1b) The impact of *de novo* missense variants on the patient transcriptomes. (a) The distribution of mean z-scores of gene expression. (b) Gene set enrichment analysis of MYRF target genes.
(PDF)

S7 Fig qPCR validation of selected genes differentially expressed between MYRF mutation carriers and other cases. The relative expression levels of six selected genes from qPCR (a) are compared with the TPM metrics of RNAseq (b).
(PDF)

S8 Fig Expression trajectories of *MYRF* and *GATA4* in mouse developing diaphragm and lung. (PDF)

S9 Fig Enrichment of damaging variants in DD/CHD genes that have not been implicated in CDH. (a) Comparing the observed vs expected number of damaging variants on DD/CHD genes after excluding known CDH candidate genes. (b) The same as (a) but using all constrained genes as the background.
(PDF)

S1 Data Demographic and clinical characteristics of cases. (XLSX)

S2 Data Full list of de novo coding variants. (XLSX)

S3 Data Putative MYRF target genes. (XLSX)

S4 Data Differentially expressed genes between *MYRF* variant carriers and other cases. (XLSX)

S5 Data Gene sets used in cross-disorder analysis. (XLSX)

S6 Data (Related to Fig4) Enriched Gene Ontology terms in the functional map. (XLSX)

S1 Table Sequencing summary. (PDF)

S2 Table (Related to Table 2) Burden of *de novo* variants in different sub-groups of patients. (PDF)

S3 Table Genes affected by multiple *de novo* functional variants. (PDF)

S4 Table Pathogenicity prediction of *MYRF de novo* missense variants. (PDF)

S5 Table LGD variants of *MYRF* in general populations. (PDF)

S6 Table Primers for qPCR validation of selected differentially expressed genes. (PDF)

S7 Table (Related to Fig 2C) Clinical information of cases carrying damaging variants in DD/CHD genes. (PDF)

S1 Text Supplementary references. (PDF)
