# Supplementary material for: De novo variants in congenital diaphragmatic hernia identify MYRF as a new syndrome and reveal genetic overlaps with other developmental disorders
Source: PLoS Genet. 2018 Dec 10;14(12):e1007822. doi: 10.1371/journal.pgen.1007822 (PMC6301721; doi:10.1371/journal.pgen.1007822)
Supplement: S1 Fig — Scatter plot and marginal histograms for mean depth and percentages of targeted regions with at least 10 or 15 reads are shown for whole-exome sequencing (a) and whole-genome sequening samples (b). (PDF) [file pgen.1007822.s001.pdf]

S1 Fig. Depth of coverage.

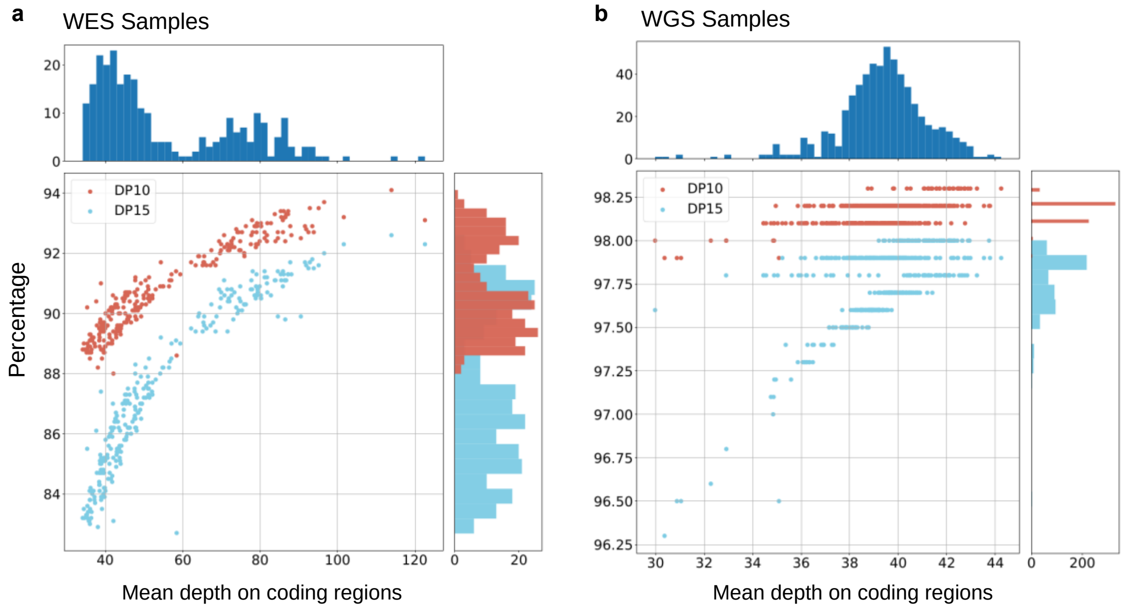

DP10/DP15 is the percentage of targeted regions that are covered by at least 10/15 unique reads. Scatter plot and marginal histograms for mean depth and DP10/DP15 are shown for 79 whole-exome sequencing (WES) trios (a) and 192 whole-genome sequencing (WGS) trios (b). Coverage statistics were calculated on the coding regions (including canonical splice sites) of GENCODE genes (v19).
