# Supplementary material for: De novo variants in congenital diaphragmatic hernia identify MYRF as a new syndrome and reveal genetic overlaps with other developmental disorders
Source: PLoS Genet. 2018 Dec 10;14(12):e1007822. doi: 10.1371/journal.pgen.1007822 (PMC6301721; doi:10.1371/journal.pgen.1007822)
Supplement: S2 Fig — Distribution of number of de novo coding variants per proband in whole-exome (a and b) and whole-genome sequenced trios (c). (PDF) [file pgen.1007822.s002.pdf]

S2 Fig. Distribution of *de novo* coding variants per proband.

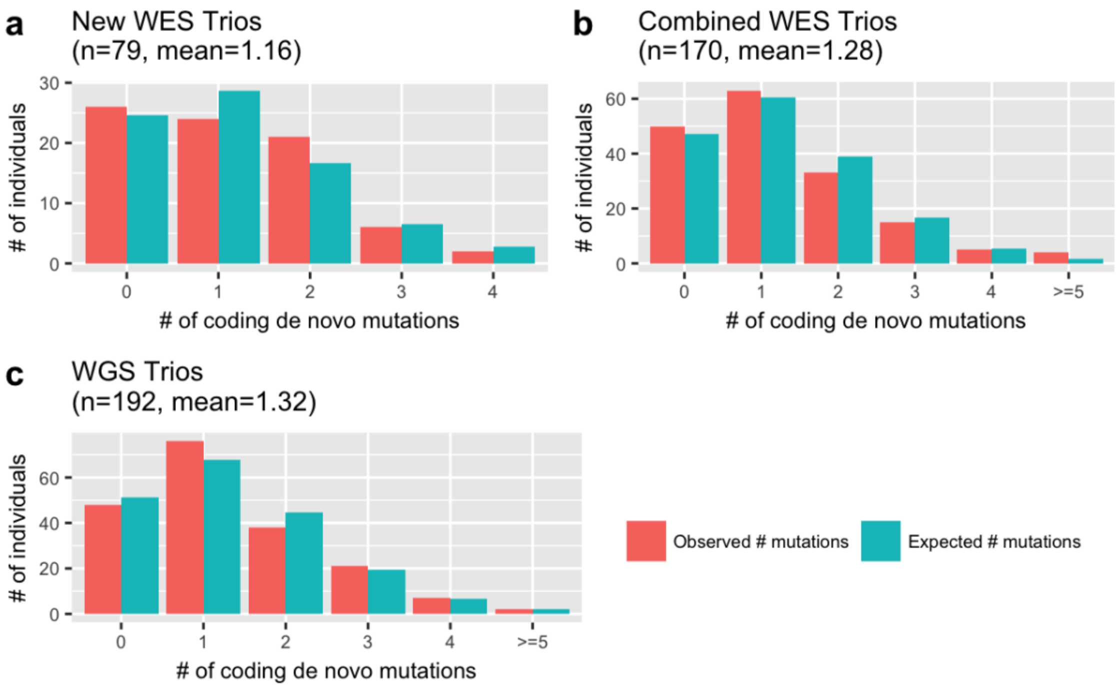

Distribution of number of *de novo* coding variants per proband in whole-exome (WES, top) and whole-genome sequencing (WGS, bottom) trios. The WES trios include 79 trios sequenced in this study, and 91 trios that were previously published. Twenty-seven published WES trios that were sequenced again by WGS were only included in the WGS analysis.

The observed number of variants were compared with the expectation assuming a Poisson distribution with mean equals to the average number of variants per proband.
