# Supplementary material for: De novo variants in congenital diaphragmatic hernia identify MYRF as a new syndrome and reveal genetic overlaps with other developmental disorders
Source: PLoS Genet. 2018 Dec 10;14(12):e1007822. doi: 10.1371/journal.pgen.1007822 (PMC6301721; doi:10.1371/journal.pgen.1007822)
Supplement: S2 Fig — Multiple sequence alignment of the DBD domain (a) and ICA domain (b) of the MYRF protein. (PDF) [file pgen.1007822.s003.pdf]

S3 Fig. Multiple sequence alignment (MSA) of MYRF protein domains.

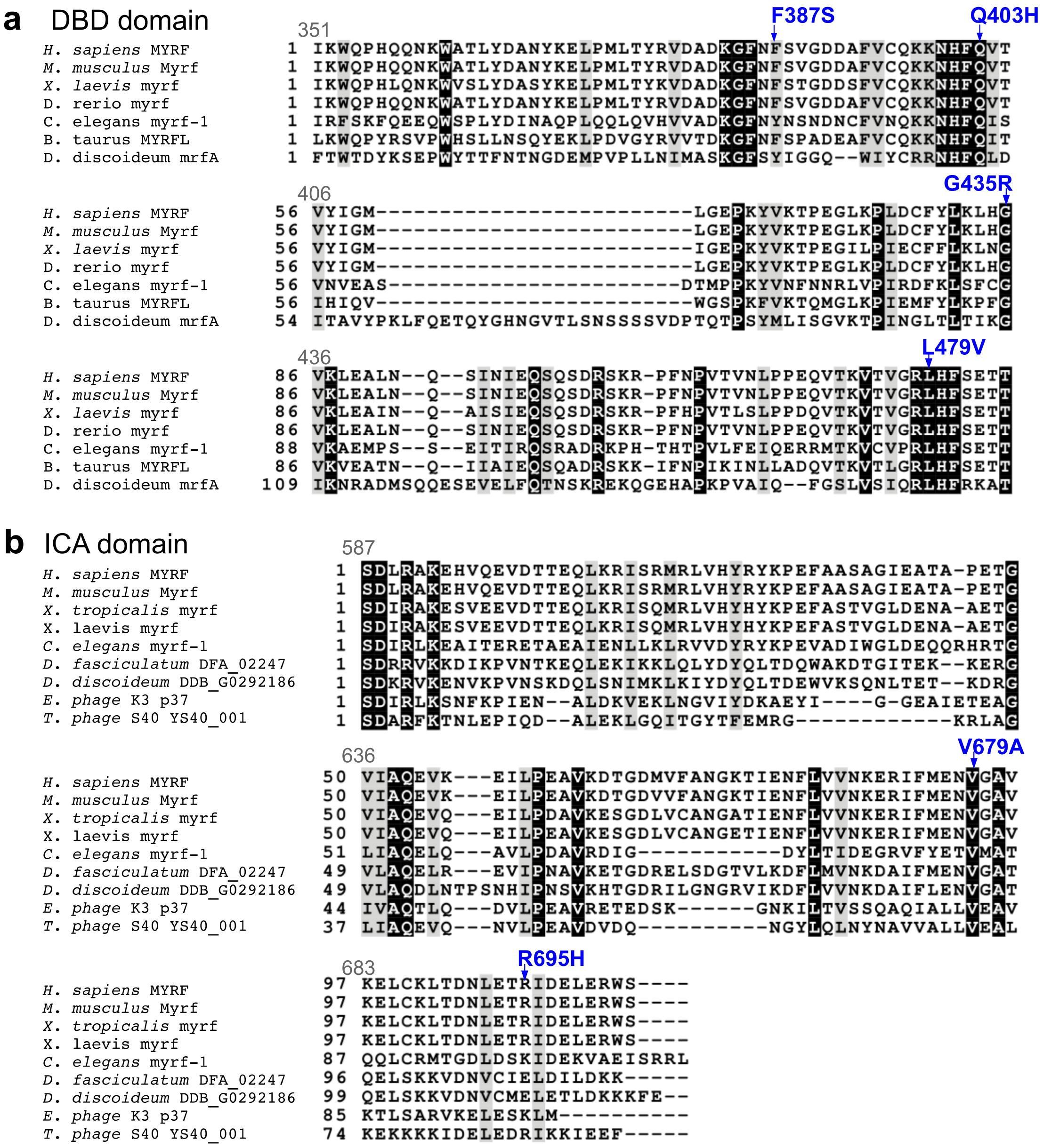

Homologous protein sequences from UniProtKB/Swiss-Prot were searched by BLASTP (https://blast.ncbi.nlm.nih.gov/Blast.cgi?PAGE=Proteins). Selected ortholog sequences on DBD and ICA domains were aligned using Clustal Omega(Sievers, Wilm et al. 2011), and the resulting MSA were then visualized by BoxShade (https://embnet.vital-it.ch/software/BOX_form.html). Residues that are completely conserved are highlighted in black; changes to residues with similar biochemical properties are shaded in gray. Amino acid substitutions that result from *de novo* missense variants are shown above the MSA. Most substitutions affect highly conserved residues.
