# Supplementary material for: De novo variants in congenital diaphragmatic hernia identify MYRF as a new syndrome and reveal genetic overlaps with other developmental disorders
Source: PLoS Genet. 2018 Dec 10;14(12):e1007822. doi: 10.1371/journal.pgen.1007822 (PMC6301721; doi:10.1371/journal.pgen.1007822)
Supplement: S4 Fig — The predicated local 3D structures of wild and mutant type proteins are shown for F387S (a), Q403H (b), G435R (c), L479R (d), and R695H (e). V679A cannot be modeled due to lack of homologues template. (PDF) [file pgen.1007822.s004.pdf]

S4 Fig. The predicted effects of *de novo* missense variants on MYRF 3D structure.

Since the sequence of MYRF DBD is almost identical between human and mouse (S3). The mouse MyRF DBD structure (PDB: 5H5P)(Zhen, Li et al. 2017) was used to assess the possible impact of missense variants.

a

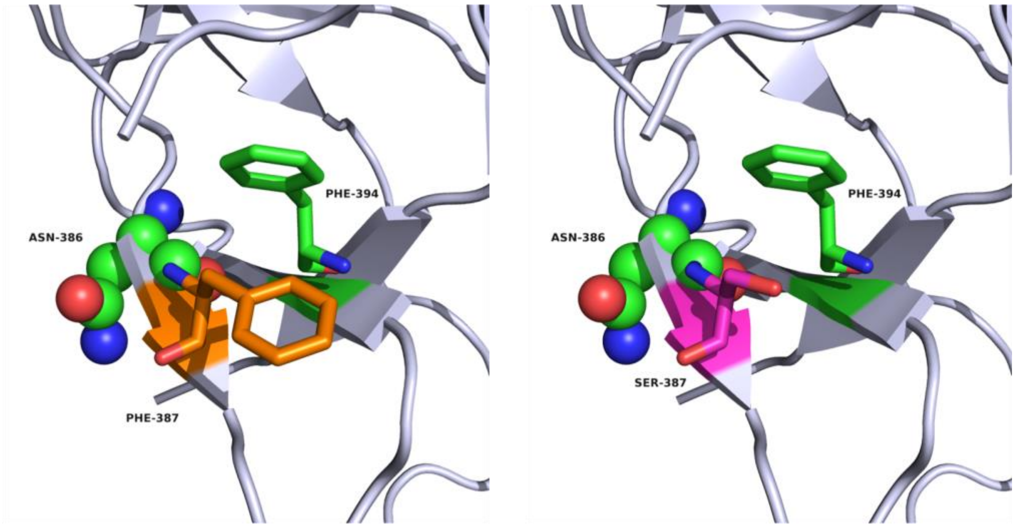

The effect of F387S substitution is to remove aromatic-aromatic interaction between F387 and F384, thus destabilize the barrel-like structure formed by nearby beta-sheets(Budyak, Zhuravleva et al. 2013). Also shown in the space filling model is N386, the neighboring residue of F387 which was predicted as one of the protein-DNA interacting sites(Chen, Zhu et al. 2018). Thus, the conformation change in the local structure caused by the substitution may also reduce the DNA binding affinity.

b

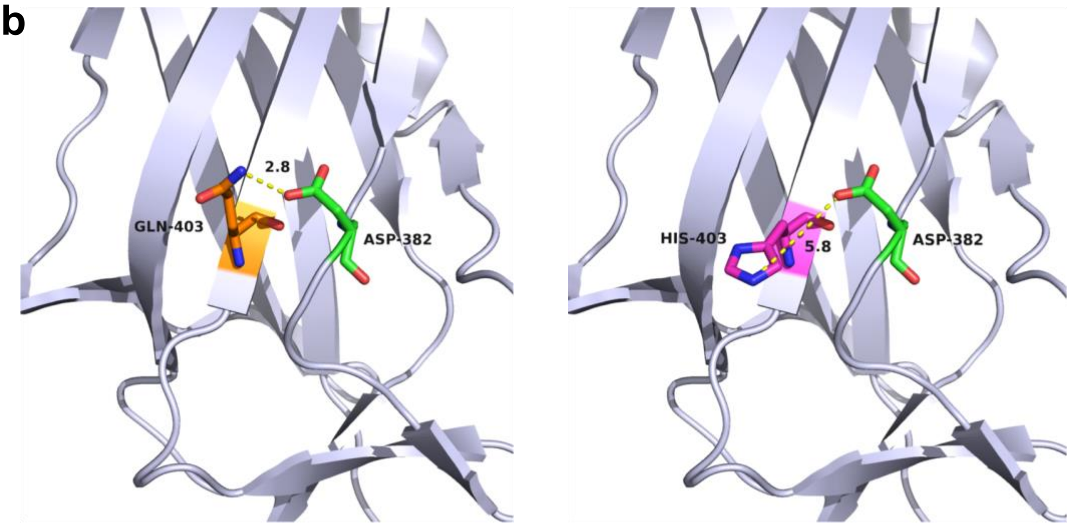

Substitution Q403H abolishes the hydrogen bond between Q403 and D382 (max distance for H-bond: 3.8 Å) and changes the surface charge distribution (Q: positive charge, H: neutral). It may influence the interaction between protomers to form trimers.

c

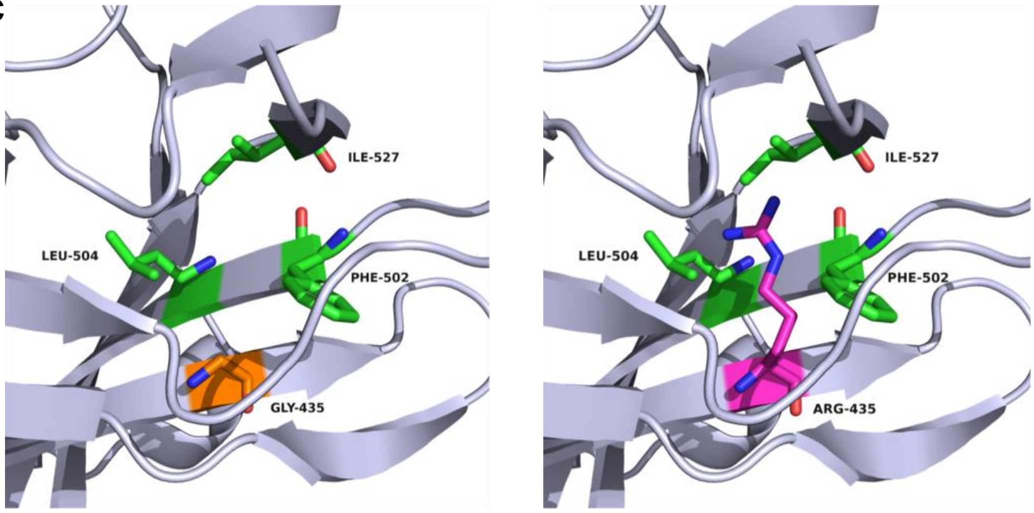

d

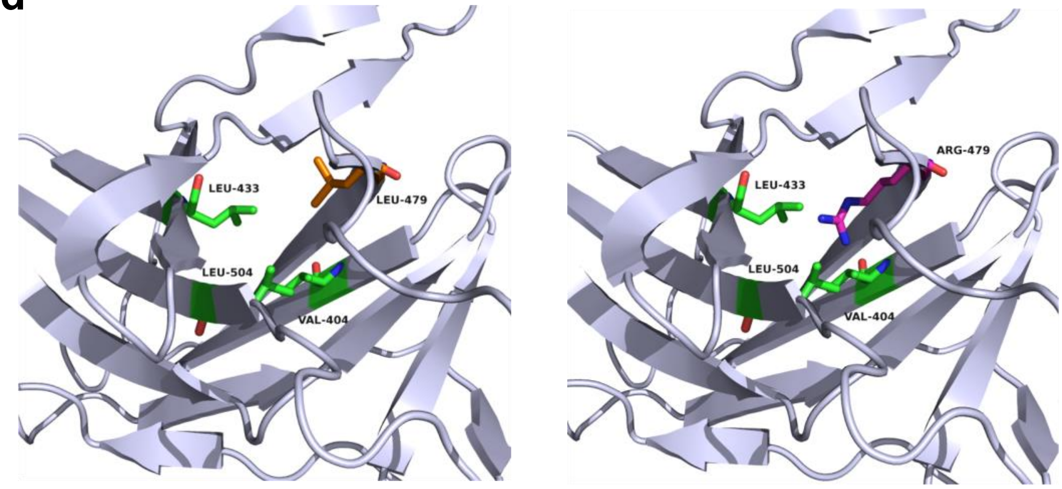

Both G435 and L479 are buried inside the hydrophobic core of MYRF DBD (shown in green are nearby hydrophobic residues). The substitution of these sites into arginine (R), a hydrophilic long side chain amino acid with positive charge, will increase the side chain clashes. The predicted changes in relative free energy (using FoldX) for Q403R and L479R are 14.3 and 8.6 Kcal/mol, respectively. Both amino acid substitutions are very likely to cause destabilization of MYRF DBD.

The structural model of human MYRF ICA domain was built by aligning the sequence with the ICA domain of bacteriophage K1F endosialidase (PDB: 3GW6)(Schulz, Dickmanns et al. 2010). Due to very distant homology, we were only confident about the alignment near C-terminal of the structure.

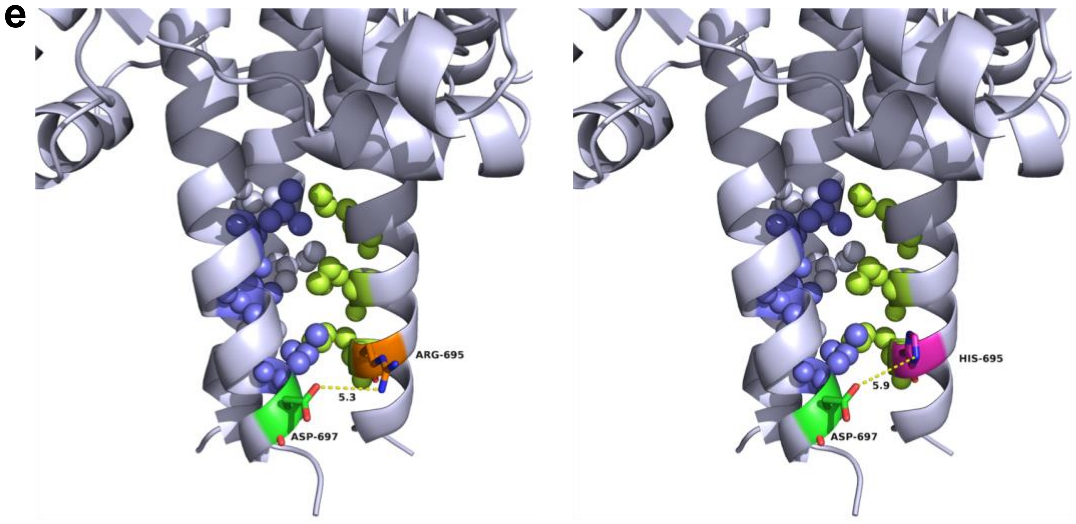

The C-terminal of the ICA domain is shown in the triplet helix form. The leucine zipper was predicted to be formed by L692, I696, and L699(Li, Park et al. 2013) which are displayed in the space filling model. The residue R695 shown in the stick model is one of the critical residue for the trimer by forming a salt bridge (max distance 5.5 Å) with D697 on the other protomer. Substitution R695H will abolish the salt bridge and may destabilize the trimer.
