# Supplementary material for: De novo variants in congenital diaphragmatic hernia identify MYRF as a new syndrome and reveal genetic overlaps with other developmental disorders
Source: PLoS Genet. 2018 Dec 10;14(12):e1007822. doi: 10.1371/journal.pgen.1007822 (PMC6301721; doi:10.1371/journal.pgen.1007822)
Supplement: S5 Fig — Principle component analysis of RNA-seq samples before (a) and after (b) removing outliers. (PDF) [file pgen.1007822.s005.pdf]

S5 Fig. Principle component (PC) analysis of RNA-seq samples.

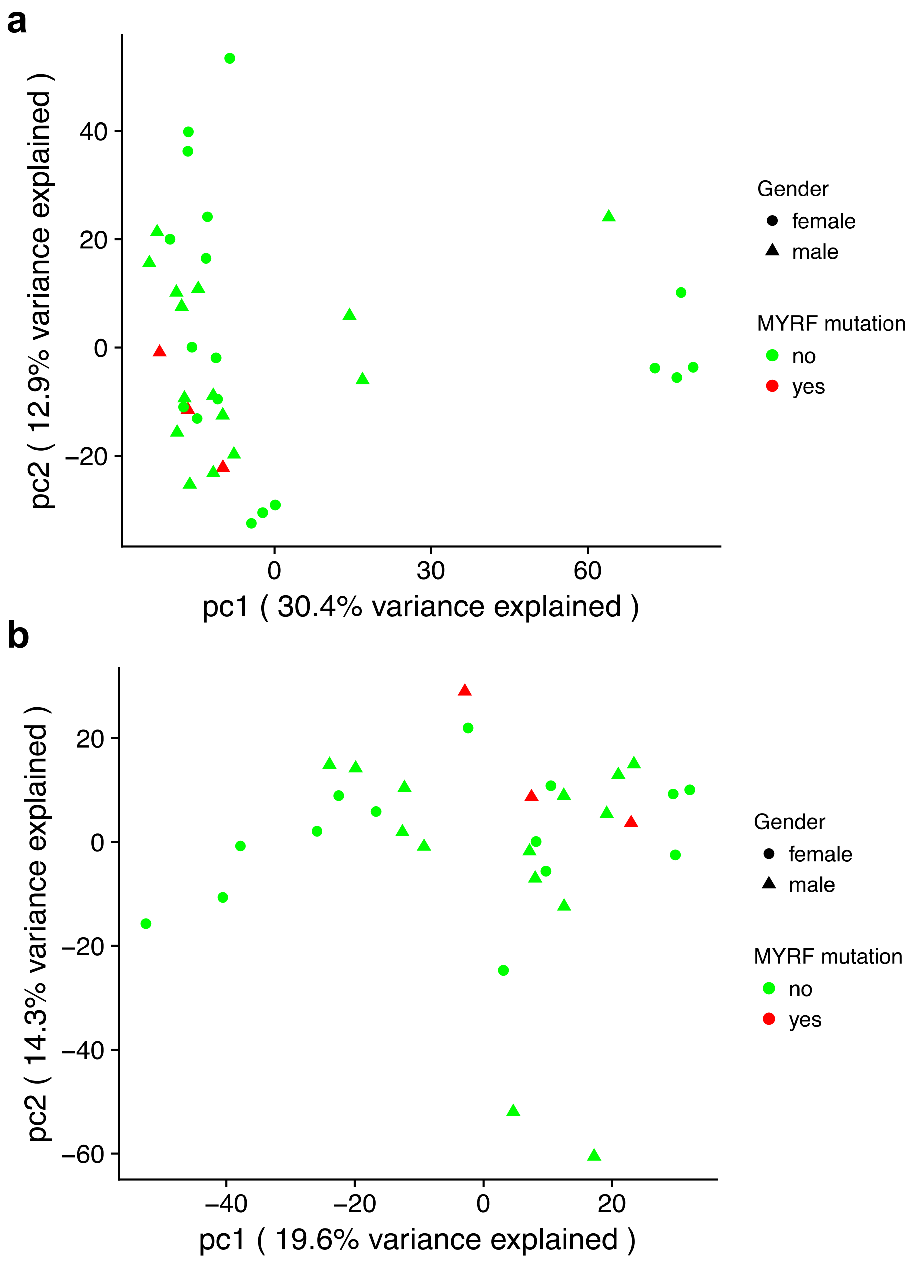

(a) The expression profile of five samples were separated from the other samples on the first two PC axis.

(b) After removing outliers, no obvious outlier can be found, and all remaining 31 samples were used in downstream analysis. Three samples from patients with *MYRF* damaging variants are indicated.
