# Supplementary material for: De novo variants in congenital diaphragmatic hernia identify MYRF as a new syndrome and reveal genetic overlaps with other developmental disorders
Source: PLoS Genet. 2018 Dec 10;14(12):e1007822. doi: 10.1371/journal.pgen.1007822 (PMC6301721; doi:10.1371/journal.pgen.1007822)
Supplement: S6 Fig — (a) The distribution of mean z-scores of gene expression. (b) Gene set enrichment analysis of MYRF target genes. (PDF) [file pgen.1007822.s006.pdf]

S6 Fig. (Related to Fig 1b and 1c) The impact of *MYRF de novo* missense variants on the patients' transcriptome.

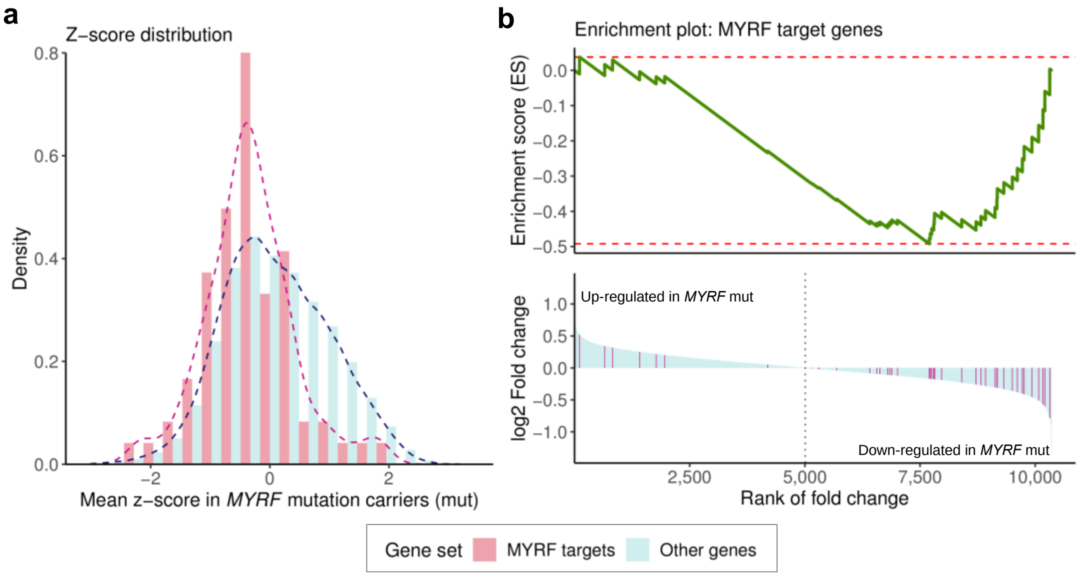

(a) The distribution of mean z-score of gene expressions in two *MYRF de novo* missense variants carriers are shown. MYRF target genes are shifted to the left side as compared with other genes (P=2.8E-5 by Kolmogorov-Smirnov test).

(b) Genes were ranked based on the differential expression level comparing two MYRF missense variants carriers against cases without *MYRF* variants MYRF target genes on average have lower ranks and majority of them are down-regulated (normalized enrichment score=-1.77, P<5.0E-4).
