# Supplementary material for: De novo variants in congenital diaphragmatic hernia identify MYRF as a new syndrome and reveal genetic overlaps with other developmental disorders
Source: PLoS Genet. 2018 Dec 10;14(12):e1007822. doi: 10.1371/journal.pgen.1007822 (PMC6301721; doi:10.1371/journal.pgen.1007822)
Supplement: S7 Fig — The relative expression levels of six selected genes from qPCR (a) are compared with the TPM metrics of RNAseq (b). (PDF) [file pgen.1007822.s007.pdf]

S7 Fig. qPCR validation of selected genes differentially expressed between MYRF mutation carriers and other cases.

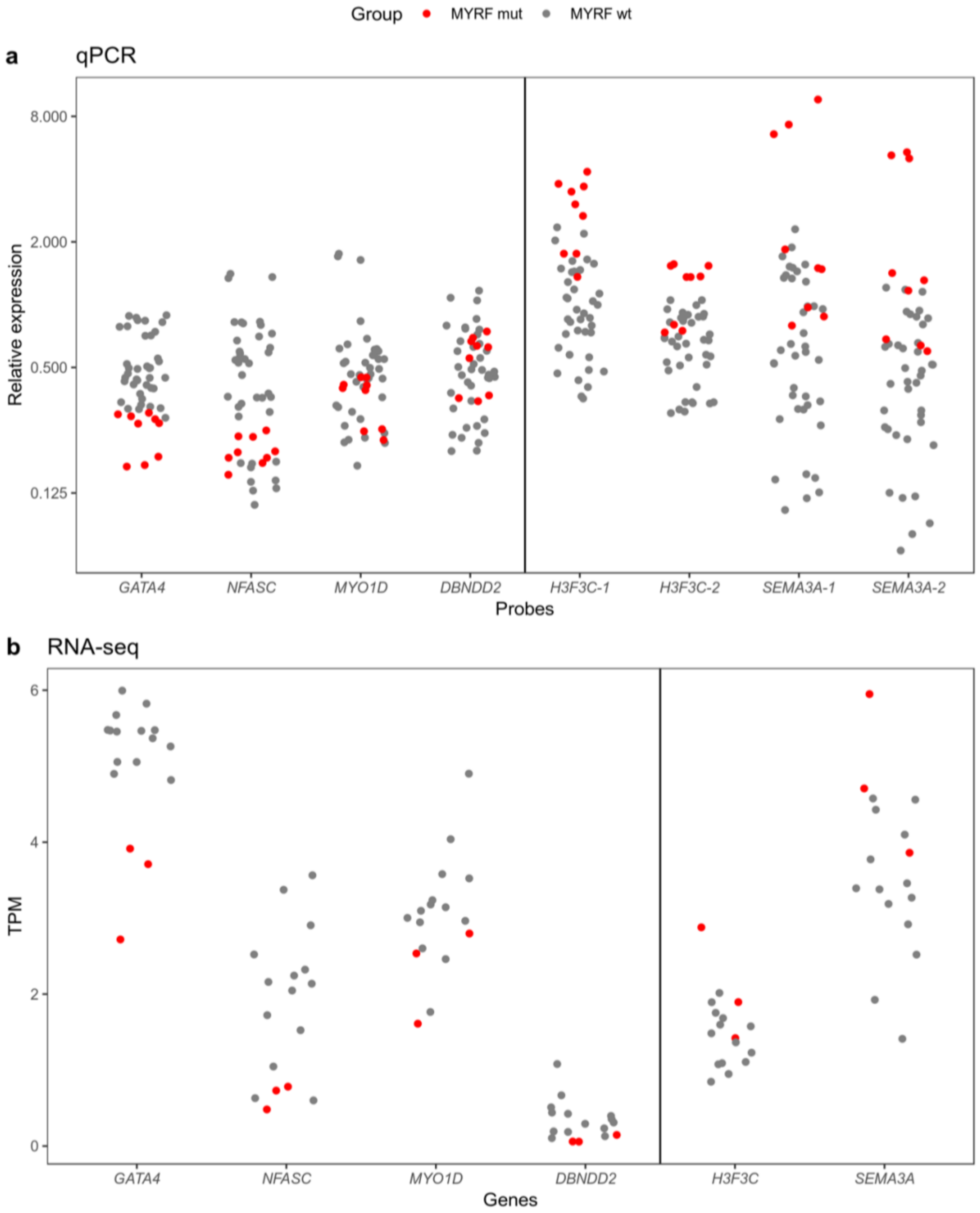

We selected six genes from the list of genes differentially expressed (DE) between *MYRF* mutation carriers and other cases (S4 Data), including four down-regulated (*GATA4*, *NFASC*, *MYO1D*, *DBNDD2*) and two up-regulated (*H3F3C*, *SEMA3A*) in *MYRF* mutation carriers. Among four down-regulated genes, the latter three are also putative MYRF target genes. For the two up-regulated genes, two primers were designed for each. We performed validation qPCR experiment in male cases (a). The results are concordant with RNAseq on male cases (b), confirming the expected trend of relative expression levels for all except *DBNDD2*. Note that *DBNDD2* is also not among the DE genes list in male-only analysis from RNA-seq (S4 Data).
