# Supplementary material for: De novo variants in congenital diaphragmatic hernia identify MYRF as a new syndrome and reveal genetic overlaps with other developmental disorders
Source: PLoS Genet. 2018 Dec 10;14(12):e1007822. doi: 10.1371/journal.pgen.1007822 (PMC6301721; doi:10.1371/journal.pgen.1007822)
Supplement: S8 Fig — (PDF) [file pgen.1007822.s008.pdf]

S8 Fig. Expression trajectories of *MYRF* and *GATA4* in mouse developing diaphragm and lung.

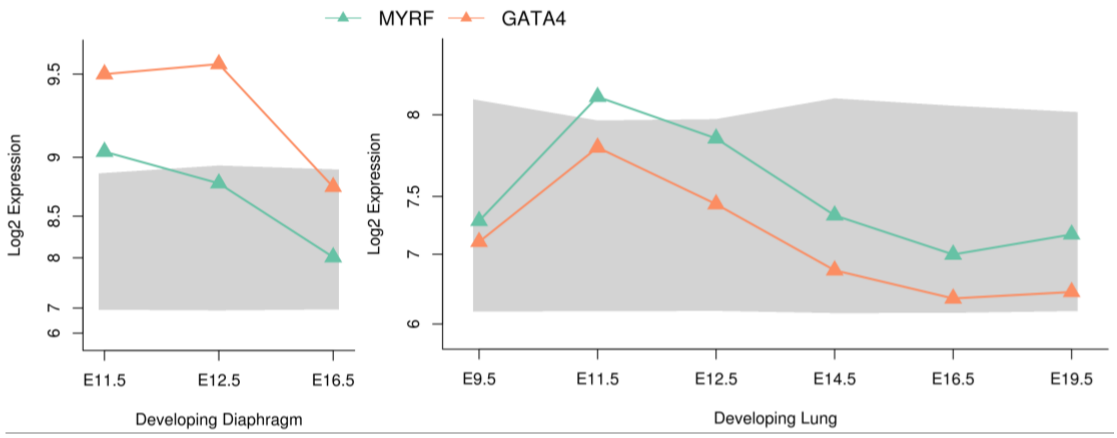

Normalized microarray gene expression levels in mouse developing diaphragm and developing lung were taken from Russell et al. 2012(Russell, Longoni et al. 2012) and Beauchemin et al. 2016(Beauchemin, Wells et al. 2016), respectively. For the lung expression data, we took the average of the three strains (C57BL/6J, A/J, and C3H/HeJ) and kept only data points in embryonic days.

In the figure above, grey areas represent 25-75% quantiles of gene expression level. It is clear that the expression of *MYRF* and *GATA4* show similar trajectories in developing diaphragm and lung and both peak at E11.5.
