# Supplementary material for: De novo variants in congenital diaphragmatic hernia identify MYRF as a new syndrome and reveal genetic overlaps with other developmental disorders
Source: PLoS Genet. 2018 Dec 10;14(12):e1007822. doi: 10.1371/journal.pgen.1007822 (PMC6301721; doi:10.1371/journal.pgen.1007822)
Supplement: S9 Fig — (a) Comparing the observed vs expected number of damaging variants on DD/CHD genes after excluding known CDH candidate genes. (b) The same as (a) but using all constrained genes as the background. (PDF) [file pgen.1007822.s009.pdf]

S9 Fig. (Related to Fig 2b) Enrichment of damaging variants in DD/CHD genes not implicated in CDH.

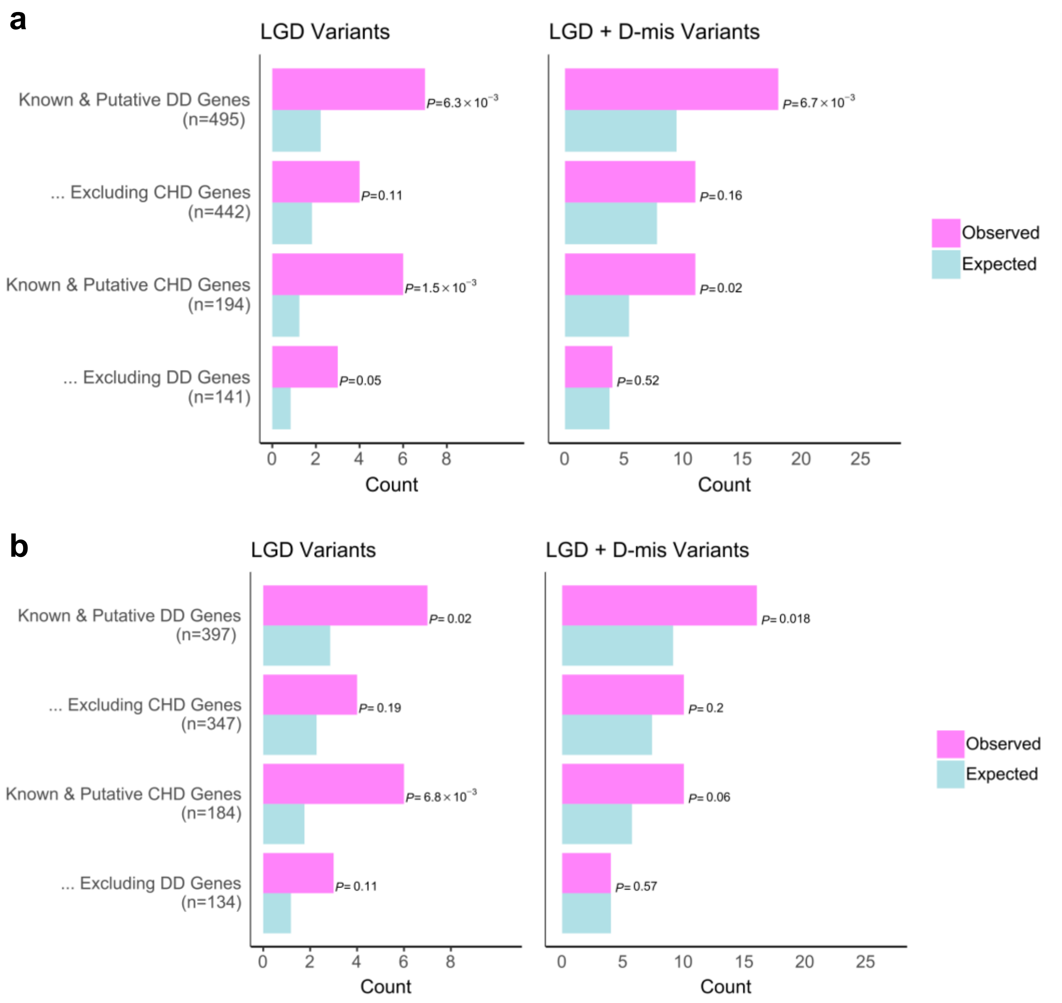

(a) To test the enrichment of *de novo* damaging variants in DD/CHD genes that have not been implicated in CDH, we excluded MYRF and 50 other CDH candidate genes(Longoni, High et al. 2014). Enrichment analysis was performed as described in Materials and Methods. (b) Since most of the overlapping genes were also constrained genes (pLI>=0.5), we also performed enrichment analysis conditional on constrained genes. *De novo* LGD variants remained significantly enriched in DD/CHD genes.
