# Supplementary material for: De novo variants in congenital diaphragmatic hernia identify MYRF as a new syndrome and reveal genetic overlaps with other developmental disorders
Source: PLoS Genet. 2018 Dec 10;14(12):e1007822. doi: 10.1371/journal.pgen.1007822 (PMC6301721; doi:10.1371/journal.pgen.1007822)
Supplement: S1 Table — (PDF) [file pgen.1007822.s016.pdf]

**S1 Tab. Sequencing summary.**

| Batch | Capture/Sequencing Protocol | Case Class | Sample Size | Total |
|---|---|---|---|---|
| DHREAMS_WES* | Aiglent SureSelect V4 or V2; 100bp paired end (PE) | Complex | 39 | 39 |
| BOSTON_WES# | NimbleGen SeqCap EZ V2; 100bp PE | Complex<br>Isolated<br>Unknown | 33<br>49<br>5 | 87 |
| DHREAMS_WES II | NimbleGen SeqCap EZ V2, 75bp PE; or Agilent SureSelect V2, 100bp PE | Complex<br>Isolated | 65<br>14 | 79 |
| DHREAMS_WGS | PCR-free whole genome sequencing; 150bp PE | Complex<br>Isolated | 42<br>150 | 192 |
| Total unique trios | | Complex<br>Isolated<br>Unknown | 149<br>208<br>5 | 362 |

A total of 362 unique proband-father-mother trios were included in the analysis.

* In 39 trios analyzed by Yu, Sawle et al. (2015), twenty-seven were also included in DHREAMS_WGS.
# In 87 trios collected by Boston Children's Hospital and Massachusetts General Hospital (Longoni, High et al. 2017), eight trios (including 4 with complex cases and 4 isolated cases) were duplicated with the DHREAMS collection. Two of them were excluded from current study due to the presence of family history, four were sequenced in the DHREAM_WES II and two were sequenced in the DHREAM_WGS.

For published trios that are not duplicates of the newly sequenced samples, we used the published de novo variants and reannotated them to GENCODE v19 coding genes. Sequence data for other trios were processed by the inhouse pipeline for variants calling.
