# Supplementary material for: De novo variants in congenital diaphragmatic hernia identify MYRF as a new syndrome and reveal genetic overlaps with other developmental disorders
Source: PLoS Genet. 2018 Dec 10;14(12):e1007822. doi: 10.1371/journal.pgen.1007822 (PMC6301721; doi:10.1371/journal.pgen.1007822)
Supplement: S2 Table — (PDF) [file pgen.1007822.s017.pdf]

S2 Tab. (Related to Table 2) Burden of *de novo* variants in different sub-groups of patients.

Case group	Variant class	Number of variants	Baseline expectation	Fold enrichment	P-value
Complex (n=149)	Silent	44	44.9	0.98	0.57
	Missense	124	103.1	1.20	0.025
	D-mis	62	38.5	**1.61**	**3.08E-04**
	LGD	23	13.5	**1.70**	**0.012**
Isolated (n=208)	Silent	63	62.7	1.01	0.50
	Missense	165	144.0	1.15	0.046
	D-mis	74	53.8	**1.38**	**5.22E-03**
	LGD	31	18.9	**1.64**	**6.52E-03**
Female (n=150)	Silent	44	45.9	0.96	0.63
	Missense	118	105.6	1.12	0.12
	D-mis	64	39.3	**1.63**	**1.85E-04**
	LGD	29	13.9	**2.09**	**2.51E-04**
Male (n=212)	Silent	66	63.2	1.04	0.38
	Missense	177	145.1	1.22	5.63E-03
	D-mis	74	54.3	**1.36**	**6.45E-03**
	LGD	28	19.0	**1.47**	**0.032**

Five patients were unknown with respect to other congenital anomalies. They were not included in either complex or isolated subgroup.
