# Supplementary material for: De novo variants in congenital diaphragmatic hernia identify MYRF as a new syndrome and reveal genetic overlaps with other developmental disorders
Source: PLoS Genet. 2018 Dec 10;14(12):e1007822. doi: 10.1371/journal.pgen.1007822 (PMC6301721; doi:10.1371/journal.pgen.1007822)
Supplement: S3 Table — (PDF) [file pgen.1007822.s018.pdf]

S3 Tab. Genes affected by multiple *de novo* LGD or missense variants.

Gene	Description	Associated Human Disorders (MIM)	pLI	*De novo* functional variants	Expected	P-value
MYRF	Myelin Regulatory Factor	-	1	1 frameshift, 3 missense	0.034	5.33E-08
WT1	Wilms Tumor 1	Denys-Drash syndrome (MIM:194080), Frasier syndrome (MIM:136680), Meacham syndrome (MIM:608978), Wilms tumor 1 (MIM:194070)	NA	2 missense	0.009	4.29E-05
SLC29A4	Solute Carrier Family 29 Member 4	-	0	2 missense	0.019	1.78E-04
WDHD1	WD Repeat And HMG-Box DNA Binding Protein 1	-	0	2 missense	0.024	2.94E-04
KIF17	Kinesin Family Member 17	-	0	2 missense	0.033	5.27E-04
TUBGCP6	Tubulin Gamma Complex Associated Protein 6	Microcephaly and chorioretinopathy (MIM:251270)	0	1 splicing, 1 missense	0.056	1.53E-03
POLE	DNA Polymerase Epsilon, Catalytic Subunit	Colorectal cancer 12 (MIM:615083), Facial dysmorphism, immunodeficiency, livedo, and short stature (MIM:615139)	0	2 missense	0.068	2.22E-03
HSPG2	Heparan Sulfate Proteoglycan 2	Dyssegmental dysplasia Silverman-Handmaker type (MIM:224410), Schwartz-Jampel syndrome (MIM:255800)	0	2 missense	0.146	9.71E-03

The observed number of de novo functional variants were compared with the expected counts in 362 trios from a baseline mutation model(Samocha, Robinson et al. 2014, Homsy, Zaidi et al. 2015). The significance of recurrence was evaluated by a one-sided Poisson test.
