# Supplementary material for: De novo variants in congenital diaphragmatic hernia identify MYRF as a new syndrome and reveal genetic overlaps with other developmental disorders
Source: PLoS Genet. 2018 Dec 10;14(12):e1007822. doi: 10.1371/journal.pgen.1007822 (PMC6301721; doi:10.1371/journal.pgen.1007822)
Supplement: S4 Table — (PDF) [file pgen.1007822.s019.pdf]

S4 Tab. Pathogenicity prediction of *MYRF de novo* missense variants.

Variants (NM_001127392.2)	CADD	MPC	SIFT	PolyPhen	MetaSVM	M-CAP
c.1160T>C:p.F387S	27.9	3.24	Deleterious	Possibly damaging	Tolerated	Deleterious
c.1209G>C:p.Q403H	27.6	2.38	Deleterious	Probably damaging	Deleterious	Deleterious
c.1303G>A:p.G435R	32	2.63	Deleterious	Probably damaging	Deleterious	Deleterious
c.1435C>G:p.L479V	23.9	2.44	Deleterious	Probably damaging	Deleterious	Deleterious
c.2036T>C:p.V679A	25.9	1.22	Deleterious	Possibly damaging	Tolerated	Deleterious
c.2084G>A:p.R695H	34	1.24	Deleterious	Probably damaging	Tolerated	Deleterious
