# Supplementary material for: De novo variants in congenital diaphragmatic hernia identify MYRF as a new syndrome and reveal genetic overlaps with other developmental disorders
Source: PLoS Genet. 2018 Dec 10;14(12):e1007822. doi: 10.1371/journal.pgen.1007822 (PMC6301721; doi:10.1371/journal.pgen.1007822)
Supplement: S5 Table — (PDF) [file pgen.1007822.s020.pdf]

S5 Tab. LGD variants of *MYRF* in general populations.

Variant	Source	Functional Consequence (NM_001127392.2)	Variant Class	Allele Count	Evaluation
11:61537790 C / CCG	ExAC	p.Pro179GlyfsTer30	Frameshift insertion	1/ 68424	False, mapping artifacts, low allele balance
11:61539012 T / TC	ExAC	p.Ser264GlnfsTer74	Frameshift insertion	78/ 120154	homo-polymer artifacts
11:61539012 TC / T	ExAC	p.Ser264AlafsTer8	Frameshift insertion	1/ 120154	
11:61537817 G / GCCCCCCC	gnomAD	p.Pro192AlafsTer38	Frameshift insertion	1/ 134254	False, mapping artifacts
11:61539110 A / AC	gnomAD	p.Ser294LeufsTer44	Frameshift insertion	2/246200	False, many indels were found in the same individual with excessive mismatches on supporting reads
11:61539113 GCC / G	gnomAD	p.Pro295ThrfsTer42	Frameshift deletion	1/246166	
11:61539116 ACCCT / A	gnomAD	p.Pro296GlyfsTer108	Frameshift deletion	1/246168	
11:61539121 G / GAGA	gnomAD	p.Trp297delinsTer	Stop gained	2/ 246160	
11:61539122 GCCTCCCC / G	gnomAD	p.Pro298ArgfsTer105	Frameshift deletion	2/246158	
11:61547305 GGTCCTCA / G	gnomAD	c.2248-5_2249del	Splice acceptor	1/245238	Real
11:61548432 G / A	CDH/CHD Parents	p.Trp829Ter	Stop gain	1/12,028	Real; p.Arg749= (synonymous) on the other isoform
11:61549043 A / G	ExAC	c.2765-2A>G	Splice acceptor	1/118730	Real
11:61550969 G / A	ExAC, gnomAD	c.3017-1G>A	Splice acceptor	2/120890	Real; does not affect the other isoform

To find LGD variants in general populations, we first extracted all loss-of-function variants of *MYRF* from gnomAD or ExAC that passed QC. (Note: although ExAC is part of gnomAD, some variants included in ExAC appear missing in gnomAD because they failed QC. So, we first took a union of the two data sets.) In addition, we also searched for the LGD variants in unaffected parents of 362 CDH cases and parents of 2645 CHD cases.

Based on Swiss-Prot, MYRF have two functional isoforms that are encoded by the transcript ENSG00000124920.9(NM_013279.2) and ENST00000278836.5 (NM_001127392.2), respectively. Functional consequences in the table above were given with respect to the Ensembl canonical transcript (ENST00000278836.5). LGD variants that affect only non-canonical transcripts were excluded. For the remaining LGD candidates, IGV snapshots were manually reviewed to judge if the variant is real.

We identified four LGD variants that passed manual review from the public and in-house database of over 120K unrelated individuals. However, two of them (p.Trp829Ter, c.3017-1G>A) affect only canonical but not the other functional isoform, and are less likely to cause complete loss of gene function. For the two remaining variants, c.2765-2A>G is a splicing variant that may truncate the protein at the position very close to C terminal end (aa. position >920), c.2248-5_2249del is a splicing region deletion whose functional consequence remains to be tested by experiment. Taken together, we could only find two splicing variants of *MYRF* in the general populations both of which had unclear functional consequence to the protein.
