# Supplementary material for: De novo variants in congenital diaphragmatic hernia identify MYRF as a new syndrome and reveal genetic overlaps with other developmental disorders
Source: PLoS Genet. 2018 Dec 10;14(12):e1007822. doi: 10.1371/journal.pgen.1007822 (PMC6301721; doi:10.1371/journal.pgen.1007822)
Supplement: S6 Table — (PDF) [file pgen.1007822.s021.pdf]

S6 Tab. Primers used for qPCR of selected differentially expressed genes

Gene	Forward	Reverse	Amplicon Size (bp)
GATA4	CGGTGCTTCCAGCAACTCCA	TGAGAACGTCTGGGACACGGA	120
DBNDD2	CCAGTCGTCTGGGATGGACA	GCAGGTTGGTGGAGGAGTCG	116
MYO1D	ACCTCGTGGAGCAACAGCAC	GCACAGAGCTTTCGGCTGGA	148
H3F3C-1	GGTGGGTCTGTTGGAAGATAC	ACTGGATGTCTTTGGGCATG	77
H3F3C-2	TGAAGCCTCATCGCTACAGG	ATCTCCCTCACCAACCTCTG	115
NFASC	GCAGGACATGCAGACCGACT	ACTCCTCGGGTGGTGAGGAC	105
SEMA3-1	GCAATAGATGGAGAACACTCTGG	ATTTGGACCTGGCACTGAG	147
SEMA3-2	GGCCAGTATCTTACACCAGAAG	GGCGTACAAGTGAGTCTGATT	114
