# Supplementary material for: De novo variants in congenital diaphragmatic hernia identify MYRF as a new syndrome and reveal genetic overlaps with other developmental disorders
Source: PLoS Genet. 2018 Dec 10;14(12):e1007822. doi: 10.1371/journal.pgen.1007822 (PMC6301721; doi:10.1371/journal.pgen.1007822)
Supplement: S7 Table — (PDF) [file pgen.1007822.s022.pdf]

S7 Tab. (Related to Fig 2c) Patients carrying damaging variants in DD/CHD genes.

Study ID	Gender	Case class	Gene	Gene set	Variant class	Cardiovascular abnormality	Neuro-developmental disorders	Other Abnormalities
49477[#]	M	Complex	*EPB41L1*<br>*POGZ*	Possible DD<br>Confirmed DD,CHD	D-mis<br>LGD		Seizures	Microcephaly, abnormal ears, micropenis, facial dysmorphism, optic nerve hypoplasia
49480[#]	F	Isolated	*NAA15*	Probable DD,CHD	LGD		NA	
01-0147[#]	F	Complex	*STAG2*	Putative CHD	LGD		Moderate intellectual disability at 5yr	Low growth hormone and precocious puberty at 9yr
01-0450[#]	F	Complex	*SIN3A*	Probable DD	LGD		Schizoaffective disorder at 17yr	Unilateral pelvic kidney, palate defect
01-0562[#]	M	Complex	*GATA6*	Confirmed CHD,DD	D-mis	ToF, 2VC	NA	Intestinal malrotation requiring Ladd surgical correction.
01-0568[#]	F	Complex	*CLCN4*	Possible DD	D-mis		Partial complex seizures at 6yr	Bilateral hip dysplasia, umbilical hernia, failure to strive
01-0634[#]	M	Complex	*PRKACB*	Putative CHD	LGD	VSD	Died	
05-0039	M	Isolated	*ATAD3A*	Confirmed DD	D-mis		Normal at 2yr	
04-0058	F	Isolated	*KDM5B*	Confirmed DD,CHD	D-mis		Lower than average at 2yr	
01-0003	M	Isolated	*MYT1L*	Possible DD	LGD		Developmental delay at 13yr	
07-0009	M	Isolated	*SRGAP3*	Possible DD	D-mis		NA	
01-0768	M	Complex	*RAF1*	Confirmed CHD,DD	D-mis		Normal at 2yr	Left extralobar pulmonary sequestration

Study ID	Gender	Case Class	Gene	Gene Set	Variant Class	Cardiovascular Abnormality	Neuro-developmental Abnormality	Other Abnormalities
06-0018	F	Isolated	*FOXP1*	Confirmed DD	LGD		Moderate development delay at 2yr	
01-0644	M	Complex	*ARID1B*	Confirmed DD,CHD	LGD	CoA	Dandy-walker malformation; died	Poorly developed scrotal sac with undescended testis, unable to palpate in the inguinal canal.
01-0552	M	Complex	*BRAF*	Confirmed CHD,DD	D-mis	2VC	NA	
07-0011	F	Isolated	*ZFPM2*	Possible DD	LGD		Died	
01-0596	M	Isolated	*EMX2*	Possible DD	LGD	RA/IVC thrombosis at birth, dilated aortic root at 5yr	Normal at 2yr	
01-0424	F	Isolated	*WT1*	Confirmed DD	D-mis		Normal at 2yr	
01-0098	F	Complex	*WT1*	Confirmed DD	D-mis		NA	Right hydronephrosis, hypotonia
02-0023	M	Complex	*PTPN11*	Confirmed CHD,DD	D-mis	Univentricular defect	Died	
04-0047	F	Isolated	*CIC*	Possible DD	D-mis		Normal at 2yr	
01-0342	F	Isolated	*HSD17B10*	Confirmed DD	D-mis		NA	
01-0113	M	Complex	*NACC1*	Confirmed DD	D-mis		Died	Pyloric stenosis
03-0013	F	Isolated	*MEIS2*	Putative CHD	D-mis		NA	
04-0059	F	Isolated	*LAMA5*	Putative CHD	LGD		Normal at 2yr	

Cases with damaging variants in *MYRF* are shown in Table 3 of the main text. Abbreviations: contraction of aorta (CoA), two-vessel cord (2VC), Tetralogy of Fallot (ToF), right atrium and inferior vena cava (RA/IVC), not evaluated for neurodevelopment outcome (NA)
#: Those cases have been reported in the previous studies(Yu, Bennett et al. 2014, Longoni, High et al. 2017).
