# Supplementary material for: De novo variants in congenital diaphragmatic hernia identify MYRF as a new syndrome and reveal genetic overlaps with other developmental disorders
Source: PLoS Genet. 2018 Dec 10;14(12):e1007822. doi: 10.1371/journal.pgen.1007822 (PMC6301721; doi:10.1371/journal.pgen.1007822)
Supplement: S1 Text — (PDF) [file pgen.1007822.s023.pdf]

Supplementary References

Beauchemin, K. J., J. M. Wells, A. T. Kho, V. M. Philip, D. Kamir, I. S. Kohane, J. H. Graber and C. J. Bult (2016). "Temporal dynamics of the developing lung transcriptome in three common inbred strains of laboratory mice reveals multiple stages of postnatal alveolar development." PeerJ **4**: e2318.

Budyak, I. L., A. Zhuravleva and L. M. Gierasch (2013). "The Role of Aromatic-Aromatic Interactions in Strand-Strand Stabilization of beta-Sheets." J Mol Biol **425**(18): 3522-3535.

Bujalka, H., M. Koenning, S. Jackson, V. M. Perreau, B. Pope, C. M. Hay, S. Mitew, A. F. Hill, Q. R. Lu, M. Wegner, R. Srinivasan, J. Svaren, M. Willingham, B. A. Barres and B. Emery (2013). "MYRF is a membrane-associated transcription factor that autoproteolytically cleaves to directly activate myelin genes." PLoS Biol **11**(8): e1001625.

Chen, B., Y. Zhu, S. Ye and R. Zhang (2018). "Structure of the DNA-binding domain of human myelin-gene regulatory factor reveals its potential protein-DNA recognition mode." J Struct Biol.

Dong, C., P. Wei, X. Jian, R. Gibbs, E. Boerwinkle, K. Wang and X. Liu (2015). "Comparison and integration of deleteriousness prediction methods for nonsynonymous SNVs in whole exome sequencing studies." Hum Mol Genet **24**(8): 2125-2137.

Homsy, J., S. Zaidi, Y. Shen, J. S. Ware, K. E. Samocha, K. J. Karczewski, S. R. DePalma, D. McKean, H. Wakimoto, J. Gorham, S. C. Jin, J. Deanfield, A. Giardini, G. A. Porter, Jr., R. Kim, K. Bilguvar, F. Lopez-Giraldez, I. Tikhonova, S. Mane, A. Romano-Adesman, H. Qi, B. Vardarajan, L. Ma, M. Daly, A. E. Roberts, M. W. Russell, S. Mital, J. W. Newburger, J. W. Gaynor, R. E. Breitbart, I. Iossifov, M. Ronemus, S. J. Sanders, J. R. Kaltman, J. G. Seidman, M. Brueckner, B. D. Gelb, E. Goldmuntz, R. P. Lifton, C. E. Seidman and W. K. Chung (2015). "De novo mutations in congenital heart disease with neurodevelopmental and other congenital anomalies." Science **350**(6265): 1262-1266.

Kircher, M., D. M. Witten, P. Jain, B. J. O'Roak, G. M. Cooper and J. Shendure (2014). "A general framework for estimating the relative pathogenicity of human genetic variants." Nat Genet **46**(3): 310-315.

Li, Z., Y. Park and E. M. Marcotte (2013). "A Bacteriophage tailspike domain promotes self-cleavage of a human membrane-bound transcription factor, the myelin regulatory factor MYRF." PLoS Biol **11**(8): e1001624.

Longoni, M., F. A. High, H. Qi, M. P. Joy, R. Hila, C. M. Coletti, J. Wynn, M. Loscertales, L. Shan, C. J. Bult, J. M. Wilson, Y. Shen, W. K. Chung and P. K. Donahoe (2017). "Genome-wide enrichment of damaging de novo variants in patients with isolated and complex congenital diaphragmatic hernia." Hum Genet **136**(6): 679-691.

Longoni, M., F. A. High, M. K. Russell, A. Kashani, A. A. Tracy, C. M. Coletti, R. Hila, A. Shamia, J. Wells, K. G. Ackerman, J. M. Wilson, C. J. Bult, C. Lee, K. Lage, B. R. Pober and P. K. Donahoe (2014). "Molecular pathogenesis of congenital diaphragmatic hernia revealed by exome sequencing, developmental data, and bioinformatics." Proc Natl Acad Sci U S A **111**(34): 12450-12455.

Love, M. I., W. Huber and S. Anders (2014). "Moderated estimation of fold change and dispersion for RNA-seq data with DESeq2." Genome Biol **15**(12): 550.

Russell, M. K., M. Longoni, J. Wells, F. I. Maalouf, A. A. Tracy, M. Loscertales, K. G. Ackerman, B. R. Pober, K. Lage, C. J. Bult and P. K. Donahoe (2012). "Congenital diaphragmatic hernia candidate genes derived from embryonic transcriptomes." Proc Natl Acad Sci U S A **109**(8): 2978-2983.

Samocha, K. E., E. B. Robinson, S. J. Sanders, C. Stevens, A. Sabo, L. M. McGrath, J. A. Kosmicki, K. Rehnstrom, S. Mallick, A. Kirby, D. P. Wall, D. G. MacArthur, S. B. Gabriel, M. DePristo, S. M. Purcell, A.

Palotie, E. Boerwinkle, J. D. Buxbaum, E. H. Cook, Jr., R. A. Gibbs, G. D. Schellenberg, J. S. Sutcliffe, B. Devlin, K. Roeder, B. M. Neale and M. J. Daly (2014). "A framework for the interpretation of de novo mutation in human disease." Nat Genet **46**(9): 944-950.
Schulz, E. C., A. Dickmanns, H. Urlaub, A. Schmitt, M. Muhlenhoff, K. Stummeyer, D. Schwarzer, R. Gerardy-Schahn and R. Ficner (2010). "Crystal structure of an intramolecular chaperone mediating triple-beta-helix folding." Nat Struct Mol Biol **17**(2): 210-215.
Sievers, F., A. Wilm, D. Dineen, T. J. Gibson, K. Karplus, W. Li, R. Lopez, H. McWilliam, M. Remmert, J. Soding, J. D. Thompson and D. G. Higgins (2011). "Fast, scalable generation of high-quality protein multiple sequence alignments using Clustal Omega." Mol Syst Biol **7**: 539.
Yu, L., J. T. Bennett, J. Wynn, G. L. Carvill, Y. H. Cheung, Y. Shen, G. B. Mychaliska, K. S. Azarow, T. M. Crombleholme, D. H. Chung, D. Potoka, B. W. Warner, B. Bucher, F. Y. Lim, J. Pietsch, C. Stolar, G. Aspelund, M. S. Arkovitz, G. University of Washington Center for Mendelian, H. Mefford and W. K. Chung (2014). "Whole exome sequencing identifies de novo mutations in GATA6 associated with congenital diaphragmatic hernia." J Med Genet **51**(3): 197-202.
Yu, L., A. D. Sawle, J. Wynn, G. Aspelund, C. J. Stolar, M. S. Arkovitz, D. Potoka, K. S. Azarow, G. B. Mychaliska, Y. Shen and W. K. Chung (2015). "Increased burden of de novo predicted deleterious variants in complex congenital diaphragmatic hernia." Hum Mol Genet **24**(16): 4764-4773.
Zhen, X., B. Li, F. Hu, S. Yan, G. Meloni, H. Li and N. Shi (2017). "Crystal structure of the DNA-binding domain of Myelin-gene Regulatory Factor." Sci Rep **7**(1): 3696.
